# Genome-wide identification and functional characterization of the two-component system gene family in petunia reveals roles in hormone signaling and stress response

**DOI:** 10.3389/fpls.2025.1721349

**Published:** 2025-12-15

**Authors:** Binbin Dai, Juntao Huo, Linxia Zhang, Peishan Zou, Miaomiao Sun, Seping Dai, Guofeng Liu

**Affiliations:** Guangzhou Collaborative Innovation Center on Science-Tech of Ecology and Landscape, Guangzhou Institute of Forestry and Landscape Architecture, Guangzhou, China

**Keywords:** petunia, two-component system (TCS), cytokinin signaling, gene expression profiling, response regulator

## Abstract

The two-component system (TCS) regulates key processes in plant growth, development, and stress response. Although TCS genes have been studied in model plants such as *Arabidopsis* and tomato, in petunia (*Petunia axillaris*), a model ornamental species, they remain poorly characterized at the genome-wide level. This study aimed to perform a comprehensive genome-wide analysis of TCS genes in petunia and investigate their expression patterns in response to hormones and abiotic stresses. A total of 74 TCS genes were identified, comprising 24 histidine kinases/histidine kinase-like (HK(L)s), 10 histidine phosphotransfers (HPs), and 40 response regulators (RRs). Comprehensive analyses of gene structures, conserved domains, and phylogenetic relationships suggested potential evolutionary divergence and functional specialization within TCS gene families. Tissue-specific expression profiles obtained via qRT-PCR revealed higher expression of TCS genes in leaves and roots. Analysis of *cis*-regulatory elements (CREs) indicated an enrichment of hormone- and stress-responsive motifs in petunia TCS promoters, with the abscisic acid (ABA)-responsive element (ABRE) being the most abundant, found in 63 genes, followed by the anaerobic response (ARE) and stress response (STRE) elements. We further analyzed response patterns to exogenous hormones (trans-zeatin (tZ) and ABA) and abiotic stresses (drought and salinity) using qRT-PCR. Compared to untreated controls, many TCS genes exhibited statistically significant up- and down-regulation (|log2FC| > 1, *p* < 0.05). These findings provide new insights into the regulatory roles of TCS genes in Petunia and identify candidate regulators for functional validation and molecular breeding to improve the floral traits and stress tolerance of ornamental plants.

## 1 Introduction

TCS, originally identified in bacteria as a mechanism for sensing and responding to environmental stimuli (Mizuno, 1997), comprises membrane-associated HK and cytoplasmic RR. It represents one of the most fundamental and widespread mechanisms of signal perception/transduction in prokaryotes (Stock et al., 2000). In eukaryotic species, including higher plants, this ancestral system has evolved into a multiple phosphotransfer pathway involving three conserved components: hybrid HKs (contain both HK and receiver (REC) domain), HPs and RRs (Loomis et al., 1997; Urao et al., 2000). The HPs mediate a multi-step His-Asp-His-Asp phosphorelay by acting as phosphohistidine intermediates to transfer the phosphoryl group from the REC domain of hybrid HKs to the REC domain of RRs. This system enables long-distance signal transmission from the membrane to the nucleus, provides more choices for signal transduction, and contributes to increasing the diversity, complexity, and accuracy of the regulation (Schaller et al., 2011).

To date, TCS genes have been identified in multiple plant species with available whole-genome sequences, including *Arabidopsis thaliana* (Hwang et al., 2002; Schaller et al., 2008), rice (*Oryza sativa*) (Pareek et al., 2006), *Lotus japonicus* (Ishida et al., 2009), *Physcomitrella patens* (Ishida et al., 2010), soybean (*Glycine max*) (Mochida et al., 2010), maize (*Zea mays*) (Chu et al., 2011), wheat (*Triticum aestivum*) (Gahlaut et al., 2014), Chinese cabbage (*Brassica rapa* ssp. *pekinensis*) (Liu et al., 2014), tomato (*Solanum lycopersicum*) (He et al., 2016a), cucumber (*Cucumis sativus* L.) and watermelon (*Citrullus lanatus*) (He et al., 2016b), melon (*Cucumis melo* L.) (Liu et al., 2020), *Cicer arietinum* (Ahmad et al., 2020), *Zizania latifolia* (He et al., 2020), *Sorghum bicolor* (Zameer et al., 2021), *Brassica napus* (Liu et al., 2023), sweet potato (*Ipomoea batatas* L.) (Huo et al., 2023), and switchgrass (*Panicum virgatum* L.) (Wu et al., 2024). As a model plant, the most extensive studies on TCS have been conducted in *Arabidopsis*, with its genome encoding 56 TCS members, comprising 17 HKs, 6 HPs, and 33 RRs (Hwang et al., 2002; Schaller et al., 2008). 17 HKs are divided into three subfamilies: AHKs, ethylene receptors, and phytochromes. AHKs subfamily includes six members, AHK1, AHK2, AHK3, AHK4 (CRE1/WOL), AHK5 (CKI2), and CKI1. Among them, AHK2, AHK3, and AHK4 are cytokinin receptors that function as positive regulators of cytokinin signal transduction, exhibiting high redundancy in regulatory functions (Nishimura et al., 2004). AHK1, AHK5 (CKI2), and CKI1 fail to bind to cytokinin and are not cytokinin receptors. Ethylene receptors exhibit divergent functions: ETR1 and ERS1 retain HK activity, whereas ETR2, ERS2 and EIN4 possess divergent HKL domains. These receptors play important roles in seed germination, leaf senescence, root and shoot elongation, fruit softening, and ripening (Ma and Dong, 2021). Phytochromes, encoded by five different genes (*PHYA*–*PHYE*), regulate various light-dependent responses such as seed germination, seedling photomorphogenesis, shade avoidance, and flowering time (Cheng et al., 2021). Among the AHPs, the five classic HPs (AHP1–AHP5) function as highly redundant positive regulators of cytokinin signaling. In contrast, AHP6 lacks the conserved histidine residue (H) required for phosphotransfer activity and known as a pseudo-HP protein. ARRs are classified into four subfamilies: type-A, type-B, type-C, and pseudo-RRs. Type-B ARRs function as transcription factors to mediate primary cytokinin responses, including activating the expression of type-A ARRs (Argyros et al., 2008; Ishida et al., 2008), thus converting external stimuli into internal signals.

As a typical representative of TCS system, the cytokinin signaling pathway is involved in many aspects of plant development, including the control of cell division and organ size. Genetic evidence reveals that gain-of-function mutations in cytokinin receptors AHK2/AHK3 enhance flowering time and floral organ size in *Arabidopsis* (Bartrina et al., 2017), cytokinin oxidase/dehydrogenase genes *ckx3 ckx5* double mutants form larger inflorescence and floral meristems (Bartrina et al., 2011). In petunia, flower-specific expression of the *Agrobacterium tumefaciens* cytokinin biosynthesis gene *isopentenyltransferase* (*ipt*) increases the flower size (Verdonk et al., 2008). Exogenous application of cytokinin significantly increases petunia corolla size (Nishijima et al., 2006), however, ABA mediates the reduction of flower size at elevated temperatures, and this reduction can be alleviated by increasing the cytokinin/ABA ratio (Sood et al., 2021). Consistently, the cytokinin receptor gene, *PhHK*, and the type-A RRs genes, *PhRR1*-*3*, were both expressed significantly higher in the petals of large-flowered varieties than in medium- and small-flowered varieties (Nishijima et al., 2011). Flower size is a key trait that determines ornamental value and influences plant evolution. Petunia, a popular ornamental plant, is also a premier model system in plant molecular biology and genetics (Vandenbussche et al., 2016). Moreover, its large floral organs and substantial natural variation in organ size confer a distinct advantage for deciphering the genetic mechanisms underlying flower size regulation. Despite its popularity, the growth, physiological performance, and flower quality are strongly affected by abiotic stresses. The TCS pathway has been demonstrated to play important roles in stress signal transduction. Studies in rice, soybean, and tomato have shown that TCS genes are involved in responses to drought, high salinity, and extreme temperature stresses (Firon et al., 2012; Sun et al., 2014; Wen et al., 2015; Wang et al., 2019).

Although *Arabidopsis* and crop TCS genes are well-characterized, petunia still lacks a genome-wide analysis, limiting insights into its TCS-mediated regulatory networks for ornamental traits and stress responses. This study presents the first comprehensive identification of TCS genes in petunia, and detailed analyses of their gene structures, conserved domains, phylogenetic relationships, and CREs were performed. Additionally, qRT-PCR was employed to evaluate their reactions to various abiotic stresses and hormone treatments. The findings will facilitate investigations into the roles of petunia TCS genes in enhancing ornamental traits, particularly flower size, and improving stress adaptation for superior physiological performance.

## 2 Materials and methods

### 2.1 Identification of TCS genes in petunia

Two different approaches were used to identify TCS genes in petunia. First, the *P. axillaris* genome assembly (Petunia_axillaris_v1.6.2) were retrieved from Solanaceae Genomics Network database (http://solgenomics.net/, accessed on 25 March 2024) (Bombarely et al., 2016). Hidden Markov Model (HMM) (Eddy, 2011) profiles of TCS characteristic domains: HisKA (PF00512), HATPase (PF02518), CHASE (PF03924), HPt (PF01627) and REC (PF00072), were downloaded from Pfam (https://pfam.xfam.org/, accessed on 27 March 2024) (Mistry et al., 2020). Then predicted proteins from *P. axillaris* genome were scanned using HMMER v3.0 (http://hmmer.org/, accessed on 9 April 2024) with E-value less than 0.01 against these HMM profiles of TCS characteristic domains. Second, the TCS protein sequences of *Arabidopsis*, tomato and tobacco (*Nicotiana tomentosiformis*) were downloaded from the *Arabidopsis* Information Resource (TAIR) database (http://www.arabidopsis.org, accessed on 8 April 2024) and the SGN (https://solgenomics.net/, accessed on 8 April 2024), respectively, and then used as queries for BLASTP (http://blast.ncbi.nlm.nih.gov, accessed on 12 April 2024) searches against the protein datasets of *P. axillaris* with E-value of 1 × 10^−5^ as the threshold. Subsequently, we compared the putative elements with *P. axillaris* and *P. hybrida* transcripts, and applied transcriptome-based corrections to rectify assembly errors in the sequences. The SMART tool (http://smart.embl-heidelberg.de/, accessed on 18 April 2024) and the NCBI CDD tools (https://www.ncbi.nlm.nih.gov/Structure/cdd/wrpsb.cgi, accessed on 22 April 2024) (Marchler-Bauer et al., 2006) were applied as the final quality control step to verify the presence of specific conserved domains and motifs in each TCS genes. Sequences lacking these essential functional domains were excluded from subsequent analysis.

### 2.2 Gene structure construction, motif analysis, and phylogenetic analysis

The exon–intron organizations of petunia TCS genes were performed using the Gene Structure Display Server (GSDS) (http://gsds.cbi.pku.edu.cn/) (Hu et al., 2014). Conserved motifs in the TCS proteins were identified by using the MEME v5.5.8 (http://meme-suite.org/tools/meme, accessed on 10 September 2024) (Bailey et al., 2015) with the optimized parameter settings: repetition number, any; minimum motif width, 6; maximum motif width, 50; maximum number of motifs, 10, 5, 10, all remaining parameters were set to their default values. Alignment of amino acid (aa) sequences was conducted with Clustal X v1.81 (Thompson et al., 2003) and results were visualized in GeneDoc software (accessed on 22 September 2024). Protein sequence identity was calculated using DNAStar Lasergene 11 software (DNASTAR Inc., Madison, WI, USA), while molecular weights and isoelectric points (PIs) were computed via ExPASy (http://web.expasy.org/compute_pi/, accessed on 24 September 2024). Subcellular localizations were predicted using DeepLoc-2.1 website (https://services.healthtech.dtu.dk/services/DeepLoc-2.1/, accessed on 25 September 2024).

Protein sequences of *Arabidopsis* (Hwang et al., 2002), rice (Ito and Kurata, 2006; Du et al., 2007; Sharan et al., 2017), maize (Chu et al., 2011), and tomato (He et al., 2016a), were obtained from previous studies. Protein sequences of petunia were shown in **Supplementary Table S5**. Phylogenetic analysis was performed using MEGA 11 with the maximum likelihood (ML) method: JTT model, 1000 bootstrap replications (Tamura et al., 2021). Then, the phylogenetic tree was visualized using the EvolView (https://www.evolgenius.info/evolview/).

### 2.3 Promoter analysis of TCS genes in petunia

Promoter regions of petunia TCS genes (defined as 2 kb upstream from the start codon) were retrieved from the *P. axillaris* genome (Petunia_axillaris_v1.6.2). These sequences were subsequently scanned for CREs using PlantCARE (http://bioinformatics.psb.ugent.be/webtools/plantcare/html/, accessed on 15 October 2024) (Lescot et al., 2002). Predictions were obtained with a default core score threshold, and a subsequent filtering step was applied to remove redundant and unannotated motifs. The identified CREs, particularly those related to hormone and stress, were cross-referenced against well-characterized consensus motifs reported.

### 2.4 Plant materials, exogenous hormones, and abiotic stresses treatments

*P. axillaris* (S26) was cultivated in growth chambers under long-day conditions (16 h light/8 h dark) at 21–22 °C with 75% relative humidity, and a light intensity of 300 μmol m^−2^ s^−1^. Three-week-old seedlings were exposed to exogenous hormones and abiotic stresses treatments. For cytokinin and ABA treatment, the seedlings were sprayed with 100 μM tZ (Mackin, Z820710) solution and 100 μM ABA (Macklin, F935019) solution, respectively. For drought treatment, the seedlings were transferred to dry filter papers. For high salt treatment, roots were immersed in nutrient solution supplemented with 200 mM NaCl. The second fully expanded leaves from the top were sampled at 0 h (control), 1 h, 3 h, 6 h, and 12 h after treatment. Three biological replicates were performed for each treatment, with each replicate defined as one individual plant. All replicates were arranged in a randomized design to ensure that environmental conditions were uniformly distributed across treatments. For the analysis of spatial expression profiles of TCS genes, different vegetative and reproductive tissues were harvested from S26, including germinating seeds (3 days after sowing), roots, stems, and leaves of adult plants (7 days after flowering), flower buds (0.5 cm) and immature fruits (7 d after pollination). All samples were frozen in liquid nitrogen immediately after collection and stored at −80°C.

### 2.5 RNA isolation and real-time PCR analysis

Total RNA was extracted by using TRIZOL reagent (Invitrogen, Germany) according to the manufacturer-recommended protocol, and the integrity of all extracted RNA samples was confirmed by 1% agarose gel electrophoresis. Then, the first-strand cDNA synthesis was carried out with 1 μg of total RNA for each sample using the PrimeScript™ RT reagent Kit with gDNA Eraser (Takara, Japan). Gene-specific primers for petunia TCS genes were designed using Primer Premier 5.0 software, and their sequences are listed in **Supplementary Table S6**. The specificity of each primer pair was examined by melting curve analysis. qRT-PCR was performed with the SYBR Premix Ex Taq (Takara, Japan) and the Roche LightCycler480 II system (Roche, USA) with the following conditions: 95°C for 15 s, followed by 40 cycles of 95°C for 15 s, 55°C for 15 s, and 72°C for 15 s. The petunia *EF1α* was employed as the reference gene for the calculation of the expression level (Mallona et al., 2010) (**Supplementary Table S6**). Three biological replicates and three technical replicates were analyzed for each sample. The 2^−ΔCt^ or 2^−ΔΔCt^ method was then employed to calculate the relative expression levels of different genes (Livak and Schmittgen, 2001), and the heatmap was generated based on the relative expression data of each gene using the pheatmap package in R software (v4.4.1).

### 2.6 Data analysis

qRT-PCR data are presented as the mean ± standard deviation (SD) from three biological replicates, each with three technical replicates. Statistical analyses were performed using IBM SPSS Statistics 22 software (IBM, New York, USA) to compare the differences between treatments. For tissue-specific expression, significant differences were analyzed by one-way ANOVA with Tukey’s *post-hoc* test (*p* < 0.05). For hormones and stresses treatments, significant differences relative to the control were determined using t-test (*p < 0.05, **p < 0.01, ***p < 0.001). Bar graphs were visualized using GraphPad Prism 7.0.

## 3 Results

### 3.1 Identification and classification of TCS genes in petunia

A total of 74 putative TCS genes were identified in the petunia genome, comprising 24 HK(L)s, 10 HPs, and 40 RRs (**Supplementary Table S2**), more than those in tomato (65 members) and *Arabidopsis* (56 members).

#### 3.1.1 Histidine kinase proteins in petunia

**Figure 1:**
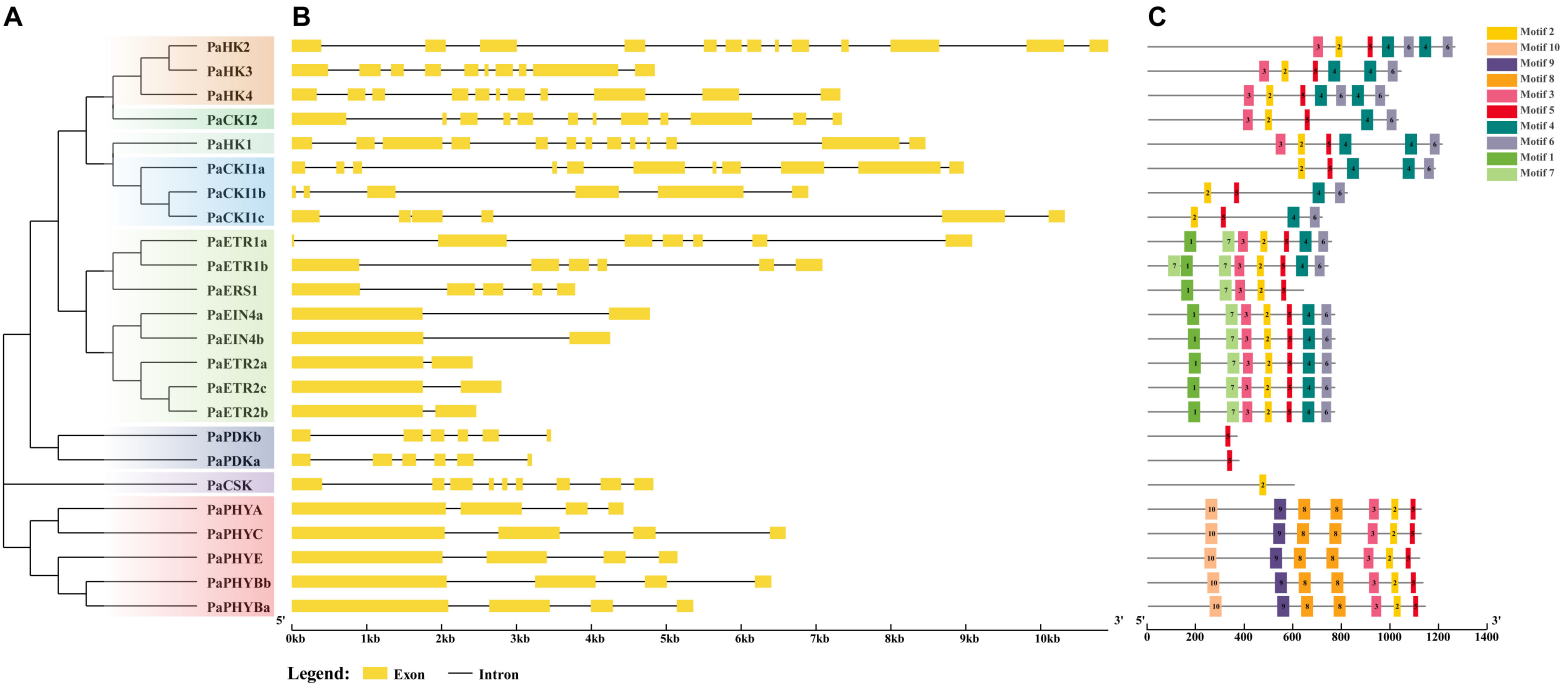
Phylogenetic relationships, gene structures, and conserved motifs of PaHK(L) genes. **(A)** Phylogenetic tree constructed based on the full-length sequences of 24 PaHK(L) proteins. **(B)** Exon–intron structure, analyzed with GSDS (yellow boxes: exons; black lines: introns). **(C)** Conserved motifs from PaHK(L) proteins are displayed in different colored boxes. The number below refers to the length of the protein.

HK proteins possess a typical conserved HK domain containing five signature motifs (H, N, G1, F, and G2) (**Supplementary Figures S1**, **S2**). The conserved histidine residue in the H motif is critical for autophosphorylation, while the remaining motifs define the nucleotide-binding cleft (Stock et al., 2000; Hwang et al., 2002). Based on the presence of the conserved HK domain, 24 PaHK(L)s could be categorized into 11 PaHKs and 13 PaHKLs (**Supplementary Figure S2**). PaHKs could be further clustered into five subgroups: three cytokinin receptors (PaHK2, PaHK3, PaHK4), three ethylene receptors (PaETR1a, PaETR1b, PaERS1), three CKI1-like (PaCKI1a, PaCKI1b, PaCKI1c), one CKI2/AHK5-like (PaCKI2), and one AHK1-like (PaHK1) (**Figure 1A**). Correspondingly, PaHKLs were identified as HKL proteins as their HK domain was divergent and also could be further categorized into four subgroups: five ethylene receptors (PaETR2a, PaETR2b, PaETR2c, PaEIN4a, PaEIN4b), five phytochrome photoreceptors (PaPHYBa, PaPHYBb, PaPHYA, PaPHYC, PaPHYE), two PDK-like (PaPDKa, PaPDKb), and one CSK-like (PaCSK) (**Figure 1A**).

PaHK2–PaHK4 contain conserved HK, HATPase, REC, transmembrane (TM), and CHASE (cyclases/histidine kinases associated sensing extracellular) domains (**Supplementary Figure S1**), shared 59.62%–67.56% identities and 63.3%–70.5% protein sequence similarities with *Arabidopsis* AHK2–AHK4 (**Supplementary Tables S2**, **S3**). The CHASE domain, essential for cytokinin perception and binding (Hwang et al., 2002), was highly conserved in PaHK2–PaHK4. Subcellular localization predictions indicated plasma membrane localization for all these three receptors, with PaHK2 also detected on the endoplasmic reticulum (ER) membrane, suggesting functional diversification (**Supplementary Table S2**). PaCKI1a–PaCKI1c share low sequence identities (33.65%–39.66%) and sequence similarities (38.1%–44.8%) with *Arabidopsis* CKI1 and lack the CHASE domain. PaHK1 contains two TM domains in the N-terminal region but also lack CHASE domain, while PaCKI2 shows no TM and CHASE domain and is predicted to localize to the cytoplasm; they shared 63.96% and 68.71% identities with AHK1 and CKI2/AHK5 in *Arabidopsis*, respectively. These structural divergences from cytokinin receptors suggest their potential involvement in non-canonical signaling pathways.

Eight ethylene receptors were identified, sharing 52.52–81.76% identities and 55.3%–82.2% similarities with their *Arabidopsis* homologs (**Supplementary Tables S2**, **S3**). These receptors contain seven conserved motifs (1–7), with the exception of PaERS1 (**Figure 1C**). PaETR1a and PaETR1b possess three TMs, along with C_2_H_4_, GAF (cGMP phosphodiesterase/adenylyl cyclase/FhlA), REC, and functional HK domain (**Supplementary Figure S1**), while PaERS1 lacks the REC domain, consistent with *Arabidopsis* ERS1, which is the only known HK protein of the non-hybrid type in plant (Grefen and Harter, 2004). PaETR2a, PaETR2b, PaETR2c, PaEIN4a, and PaEIN4b exhibit divergent HK domain and 3–4 TMs (**Supplementary Figures S1**, **S2**). They all contain one intron and two exons (**Figure 1B**), this similar exon-intron architecture was also found in *Arabidopsis* homologs. In PaEIN4a and PaEIN4b, the histidine (His) of H motif was replaced by asparagine (Asn), while PaETR2a, PaETR2b, and PaETR2c have conserved His residue but miss the other four signature motifs. Thus, these receptors may not act as His protein kinases and are thought to function as serine/threonine kinase, consistent with *Arabidopsis* ETR2, ERS2, and EIN4. This indicates HK activity may not be required for ethylene receptor function in plants. Five phytochromes (PaPHYBa, PaPHYBb, PaPHYA, PaPHYC, PaPHYE) shared 62.41%–78.77% identities and 64.2%–79.1% similarities with their *Arabidopsis* homologs, all containing PHY (phytochrome-specific GAF-related), GAF, and PAS (Period/ARNT/Single-minded) domains (**Supplementary Figure S1**), forming a PAS-GAF-PHY tri-domain in the N-terminal (Nagatani, 2010). The motif 2, 3, 5 and 8–10 are conserved in all five genes (**Figure 1C**). These phytochromes lack TM domains and show subcellular localization to the cytoplasm. PaPDKa and PaPDKb contain conserved HK domains, but PaCSK only contain part of HK domain.

#### 3.1.2 HP proteins in petunia

**Figure 2:**
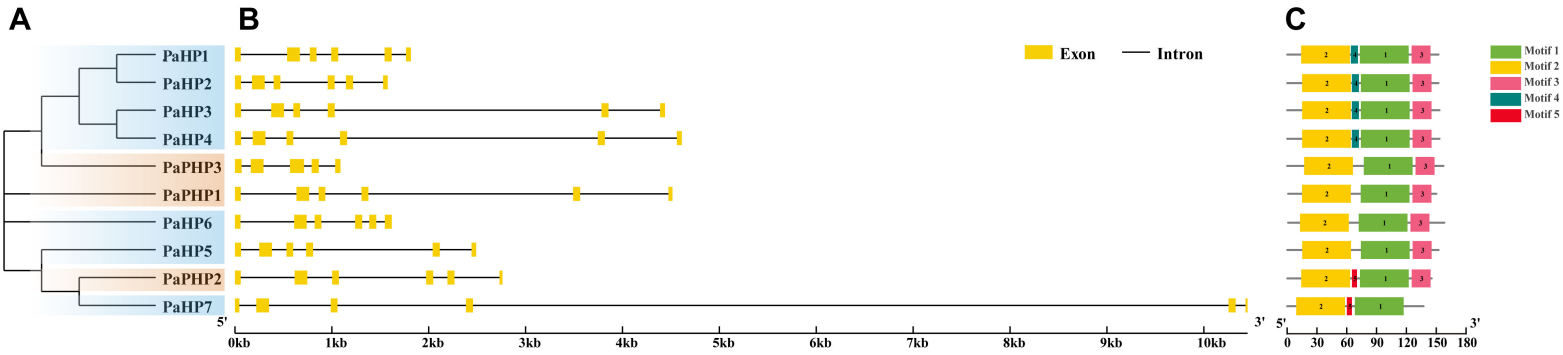
Phylogenetic relationships, gene structures, and conserved motifs of PaHP genes. **(A)** Phylogenetic tree constructed based on the full-length sequences of 10 PaHP proteins. **(B)** Exon–intron structure, analyzed with GSDS (yellow boxes: exons; black lines: introns). **(C)** Conserved motifs from PaHP proteins are displayed in different colored boxes. The number below refers to the length of the protein.

Phylogenetic and sequence alignment analyses identified 10 HP proteins in petunia, classified into two subgroups based on the presence of a conserved His phosphorylation site: 7 authentic HPs (PaHP1–7) and 3 pseudo-HPs (PaPHP1–3) (**Figure 2A**). All seven PaHPs contain the HPt domain with a signature motif of XHQXKGSSXS (**Supplementary Figure S3**), the conserved His residue is essential for phosphotransfer activity. In contrast, three pseudo-HPs exhibit substitutions at the critical His residue in the HPt domain: isoleucine (Ile) in PaPHP1, tyrosine (Tyr) in PaPHP2, and Asn in PaPHP3 (**Supplementary Figure S3**). These pseudo-HP proteins cannot function as a phosphotransfer protein, suggesting a role as competitive inhibitors of authentic HPs.

Gene structure analysis showed that most PaHP genes possess six exons and five introns, except *PaPHP3*, which has five exons and four introns (**Figure 2B**). All PaHPs contain two conserved motifs (motifs 1 and 2) (**Figure 2C**). Sequence analysis revealed that PaPHP1 and PaHP6 show low identities (32.33% and 40.56%) and similarities (29.9% and 40.0%) with their *Arabidopsis* homologs, other PaHPs share a high degree sequence identities (62.96%–80.00%) and similarities (62.0%–79.4%) with their counterparts in *Arabidopsis* (**Supplementary Tables S2**, **S3**). Notably, PaPHP1–2 show higher sequence homology to the authentic AHP4 than the pseudo-AHP6. PaPHP3 shares a high sequence identity (80.00%) to authentic AHP6 in *Arabidopsis* despite the loss of the conserved Hpt domain. Analysis of the physicochemical properties showed that PaHPs ranged from 136 to 156 aa in length, with predicted molecular weights of 15.88 to 18.21 kDa and theoretical isoelectric points (pI) spanning from 4.69 to 8.40. All were predicted to be localized in the cytoplasm and nucleus, except PaPHP3, only in the cytoplasm (**Supplementary Table S2**).

#### 3.1.3 RR proteins in petunia

**Figure 3:**
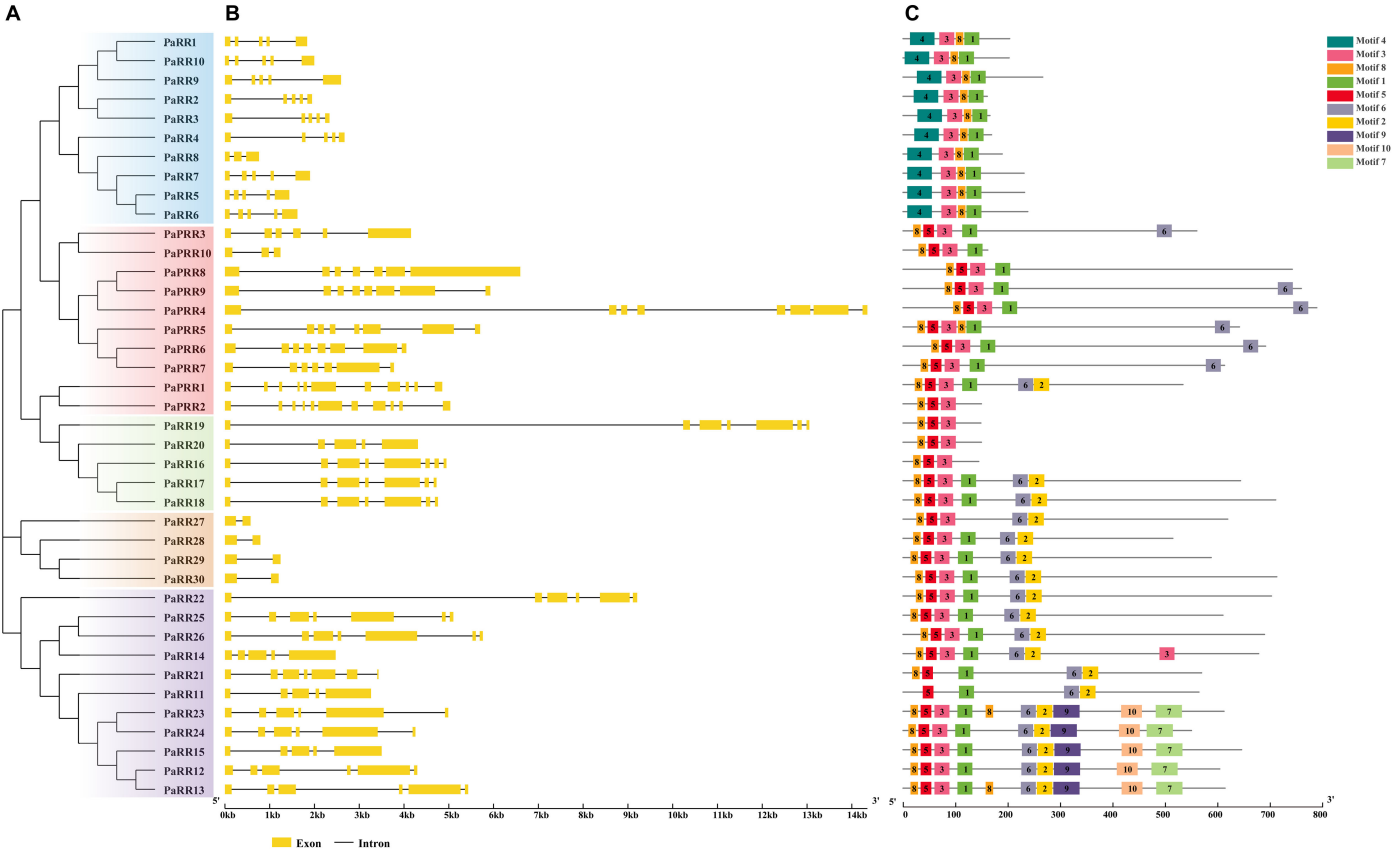
Phylogenetic relationships, gene structures, and conserved motifs of PaRR genes. **(A)** Phylogenetic tree constructed based on the full-length sequences of 40 PaRR proteins. **(B)** Exon–intron structure, analyzed with GSDS (yellow boxes: exons; black lines: introns). **(C)** Conserved motifs from PaRR proteins are displayed in different colored boxes. The number below refers to the length of the protein.

PaRRs could be divided into four subfamilies: type-A RRs (PaRR1–10), type-B RRs (PaRR11–26), type-C RRs (PaRR27–30), and pseudo-RRs (PaPRR1–10) (**Figure 3A**). Most RRs contain a REC domain (**Supplementary Figure S4**), and two conserved aspartate (D) and lysine (K) residues (D-D-K) are the key feature (**Supplementary Figure S5**), which is essential for phosphoryl group transfer during signal transduction.

All type-A RRs contain four introns, with the exception of *PaRR8*, which has only two (**Figure 3B**). The encoded proteins range from 157 to 261 aa in length, corresponding to predicted molecular weights of 17.49 to 28.65 kDa. Predictions of subcellular localization indicated that the majority of these proteins localize in the nucleus, whereas two relatively divergent proteins, PaRR2 and PaRR3, localize in both the nucleus and cytoplasm. Furthermore, Type-A RRs possess four conserved motifs (motif 1, 3, 4 and 8) (**Figure 3C**), and only contain one conserved REC domain along with short C-terminal extensions, share a high degree sequence identities (59.75% to 77.27%) and similarities (61.8% to 73.3%) with their counterparts in *Arabidopsis* (**Supplementary Tables S2**, **S3**). In contrast, type-B RRs are larger proteins, with the aa sequence length ranging from 505 to 700, and corresponding to molecular weights of 56.38 to 76.32 kDa. These type-B RRs possess four conserved motifs (motif 1, 2, 5 and 6), one REC domain at their N-terminal and one Myb-like DNA-binding domain at their C-terminal along with long C-terminal extensions, all were predicted to localize in the nucleus. Type-C RRs, though structurally similar to type-A RRs, have very low homology with type-A RRs based on phylogenetic analysis. All contain only one intron (**Figure 3B**) and encode short proteins (141–146 aa) with low molecular weights (15.75–16.46 kDa), which are predicted to be localized in both the cytoplasm and nucleus.

Compared to real RR, PRRs lack the invariant phospho-accepting aspartate site, named pseudo-REC domain, which does not participate in cytokinin signal transduction. Petunia PRR subfamily could be further classified into two groups: two type-B PRRs (PaPRR1 and PaPRR2) and eight clock PRRs (PaPRR3–PaPRR10). PaPRR1 and PaPRR2 process a pseudo-REC domain and a Myb domain, sharing 48.67%–48.72% sequence identities and 58.1%–59.4% sequence similarities with *Arabidopsis* APRR2. Eight clock PRR proteins (PaPRR3–PaPRR10) contain pseudo-REC domain at the N-terminus and CCT (CONSTANS, CO-like, and TOC1) domain at the C-terminus, except for PaPRR8 and PaPRR10 which lack the CCT domain (**Supplementary Figure S4**). All PRRs are predicted to be localized in the nucleus.

### 3.2 Phylogenetic analysis of petunia TCS proteins

To elucidate the evolutionary relationships of TCS genes across angiosperms, unrooted ML phylogenetic trees were constructed respectively using 97 HK(L)s, 33 HPs, and 182 RRs from petunia, *Arabidopsis*, tomato, rice, and maize. The trees were generally reliable, as the bootstrap values for most nodes approached or exceeded 50, with the majority exceeding 90.

**Figure 4:**
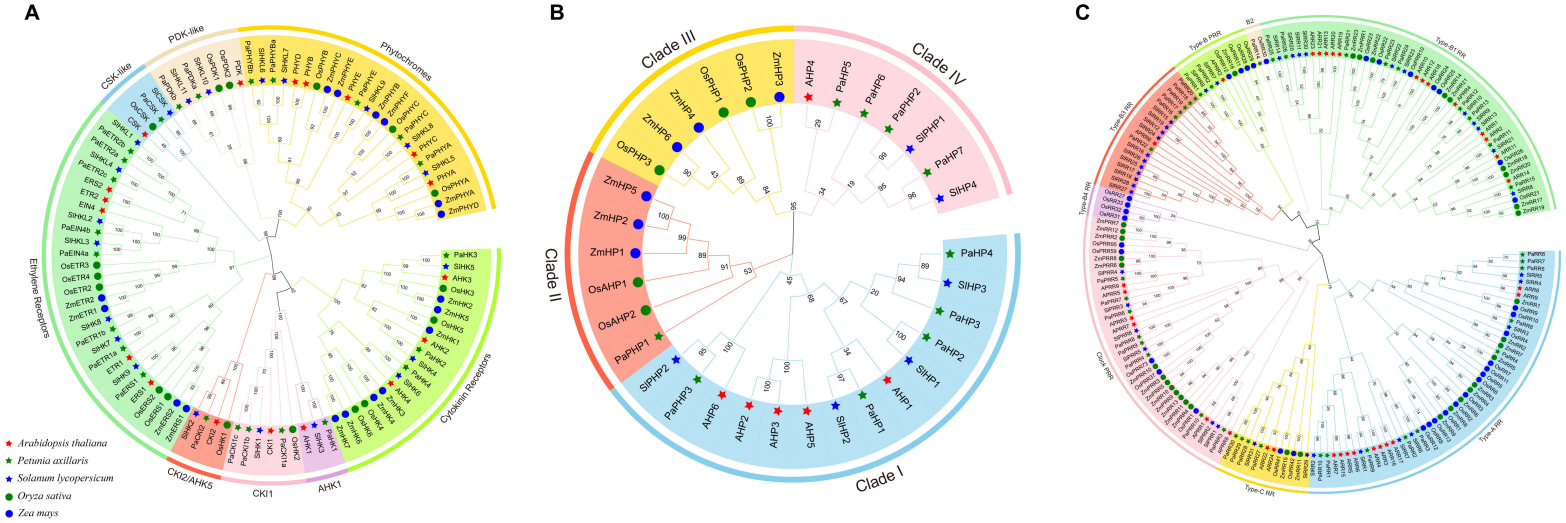
Phylogenetic relationships of HK(L)s **(A)**, HPs **(B)** and RRs **(C)** in *Arabidopsis*, rice, maize, tomato, and petunia. Different subfamilies are represented by different colors. Bootstrap values from 1000 replicates are shown at key nodes.

The results showed that the 97 HK(L) proteins were clustered into eight conserved subfamilies, namely, cytokinin receptors, CKI1, AHK1, CKI2/AHK5, PDK-like, CSK-like, ethylene receptors, and phytochromes (**Figure 4A**). Interestingly, most subfamilies of the HK(L)s are expanded in higher plants. In contrast, the cytokinin receptors subfamily was always restricted to two to five members. Within the ethylene receptor subfamily, two distinct groups emerged, one group show HK activity and another group contain divergent histidine-kinase-like (HKL) domains. Notably, maize HKs are restricted to cytokinin receptors, ethylene receptors, and phytochromes, which is similar to previous research results (Chu et al., 2011).

HPs from the five species were clustered into four distinct clades (**Figure 4B**): clade I and clade IV exclusively comprise dicot HPs (petunia, *Arabidopsis*, and tomato); clade II contains five monocot HPs and one petunia HP (*PaPHP1*); clade III exclusively contains monocot HPs (rice and maize). It can be clearly seen that HPs from the dicot species exhibit closer phylogenetic relationships, whereas HPs from the monocot species (rice and maize) are more similar to each other.

For the RRs, all members from the five species were grouped into five clades, namely type-A RRs, type-B RRs, type-C RRs, type-B PRRs, and clock PRRs (**Figure 4C**). Type-A RRs exhibited remarkably close evolutionary relationships across species. As the largest subgroup, type-B RRs were further subdivided into four distinct clusters: type-B1 RRs include members from all five species; type-B2 RRs contain two members (*PaRR14* and *OsRR30*); type-B3 RRs exclusively comprise dicot RRs; and type-B4 RRs include only four RRs from rice. Notably, although type-B PRRs and clock PRRs are both members of the PRR family, phylogenetic analysis indicated that type-B PRRs are more closely related to type-B RRs than to clock PRRs.

### 3.3 Analysis of CREs in the putative promoter regions of TCS genes in petunia

CREs play important roles in plant development and response to the environment (Marand et al., 2023). To investigate the regulatory potential of petunia TCS genes, we analyzed CREs within the promoter regions (2 kb upstream of transcriptional start sites), which contain the majority of conserved and functionally relevant CREs for the TCS genes. These promoter regions were enriched with diverse CREs associated with plant growth, development, phytohormone responses, and abiotic/biotic stress adaptation (**Supplementary Table S4**).

**Figure 5:**
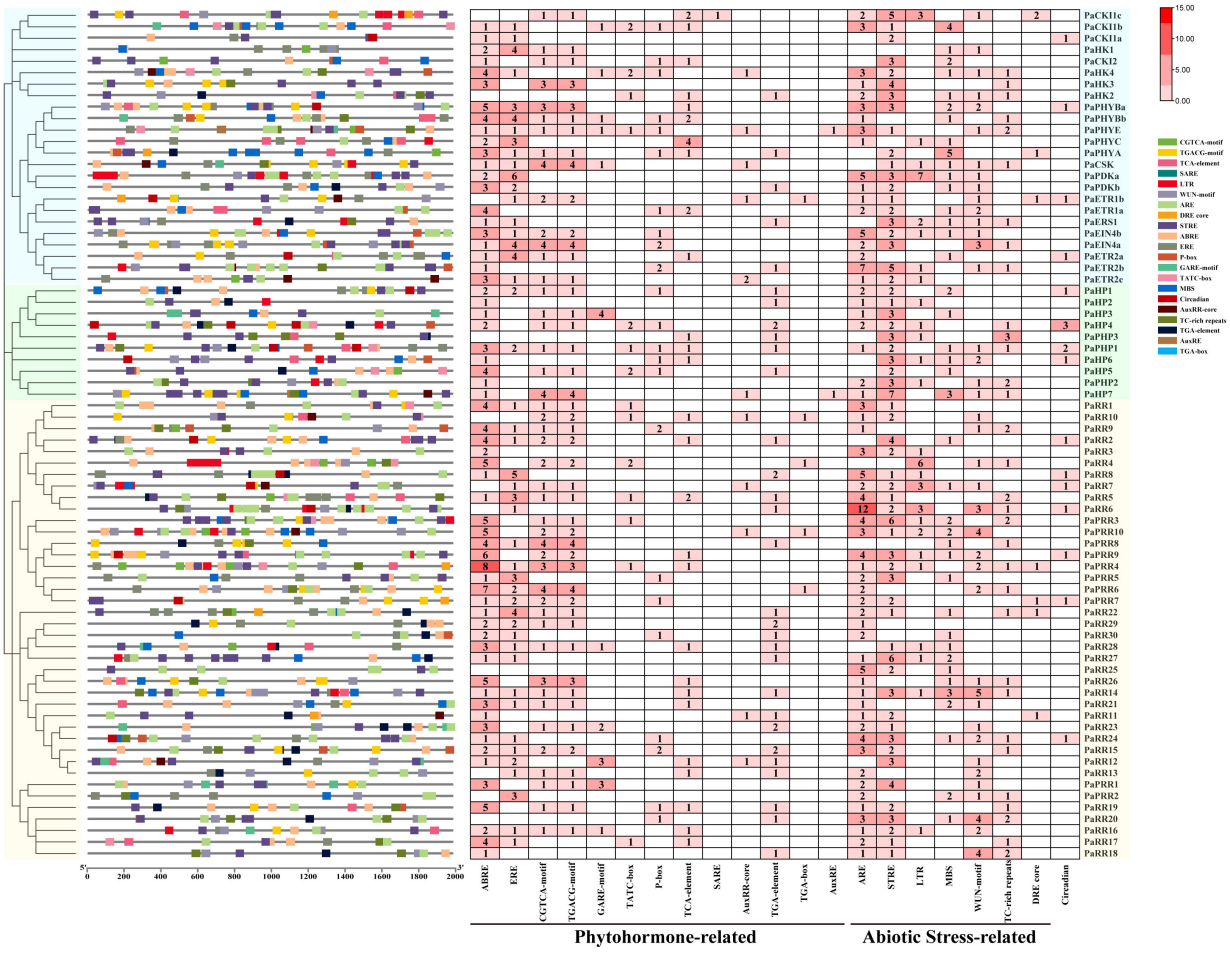
Distribution (left) and number (right) of CREs identified in putative promoter regions of TCS genes in petunia. The numbers in the heatmap represent the quantity of elements.

We further analyzed the CREs related to phytohormones and abiotic stresses (**Figure 5**). Among phytohormone-related CREs, those responsive to ABA (ABRE), ethylene (ERE), and MeJA (CGTCA-motif and TGACG-motif) were identified in the promoter regions of 63, 46, and 46 petunia TCS genes, respectively, accounting for approximately 29%, 15.6%, and 27.9% of the total number of phytohormone-related CREs. In addition, gibberellin-responsive elements (GARE-motif, TATC-box, P-box), salicylic acid-related elements (TCA-element, SARE), and auxin-responsive elements (AuxRE, AuxRR-core, TGA-element, TGA-box) were also widely distributed. Furthermore, abiotic stress-related CREs were notably abundant, including motifs associated with anaerobic induction (ARE), drought-inducibility (MBS), low-temperature (LTR), wounds (WUN-motif), heat stress (STRE), defense and stress responsiveness (TC-rich repeats), as well as drought and high-salinity stress responsive (DRE core). Among these stress-related motifs, both ARE and STRE were each detected in the promoters of 60 TCS genes, accounting for approximately 27% and 27.9% of total stress-responsive elements, respectively. These findings are consistent with reports in other plant species, where CREs related to ABA, MeJA, and heat stress responses are also abundant (He et al., 2016a, b; Liu et al., 2023). Moreover, CREs involved in developmental and physiological regulation were identified, including those associated with circadian rhythms (circadian), seed-specific expression (RY-element), cell cycle regulation (MSA-like), and meristem expression (CAT box). Light-responsive elements are detected as the most abundant category, such as AE box, G-box, Sp1, GATA motif, ACE, Box 4, G-box, the TCCC and TCT motifs.

The prevalence of specific CREs implies that petunia TCS genes are involved in hormone signaling and abiotic stress adaptation, particularly in ABA response, anaerobic induction, and heat stress.

### 3.4 Expression profiles of TCS genes in petunia

**Figure 6:**
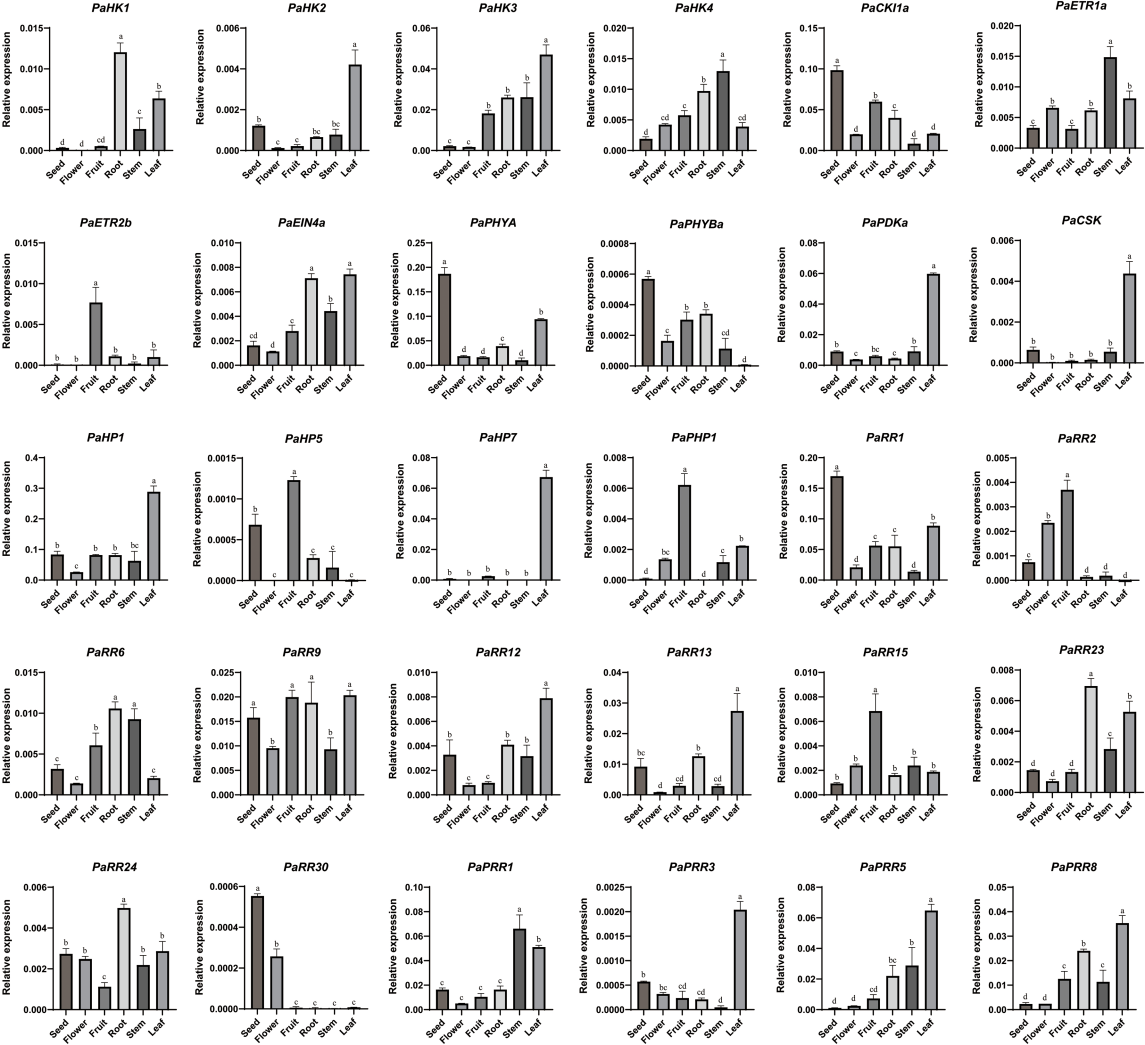
Organ-specific expression profiles of TCS genes in petunia. The relative expression level was normalized to the petunia *EF1α* gene and quantified using the 2^−ΔCT^ method. Data represent mean ± SD (standard deviation) values from three biological replicates per tissue. Significant differences (letters a-d above the bars) among tissues were analyzed by one-way ANOVA with Tukey’s *post-hoc* test (*p* < 0.05).

To better understand the potential roles of TCS genes in petunia growth and development, tissue-specific expression profiles of 30 TCS genes were analyzed across germinating seeds, roots, stems, leaves, flower buds, and young fruits (**Figure 6**). Overall, most TCS genes were highly expressed in leaves and roots but showed lower expression in flowers and seeds. Among them, *PaHK2*, *PaCSK*, *PaPH5*, *PaRR2*, and *PaPRR3*, particularly *PaPHYBa* and *PaRR30*, showed very low expression levels (less than 0.005 relative to the reference gene *PaEF1α*) across all examined tissues. In contrast, *PaCKI1a*, *PaPHYA*, *PaHP1*, *PaRR1*, *PaRR9*, *PaPRR1* were ubiquitously expressed in almost all tissues, while the remaining genes displayed distinct tissue-specific patterns. This revealed significant variability in transcript abundance among TCS genes in petunia. Within the HK family, *PaHK1–PaHK4* were more highly expressed in vegetative tissues than in reproductive organs. Specifically, *PaHK1* showed root-predominant expression, *PaHK2* and *PaHK3* exhibited leaf-predominant expression, and *PaHK4* was predominantly expressed in stems and roots. Consistently, the *Arabidopsis* homologs, *AHK2–4*, function in shoot growth, leaf senescence, germination, and root development (Nishimura et al., 2004; Riefler et al., 2005). *PaCKI1a* displayed broad tissue expression but enriched in seeds and fruits. *PaETR2b* was highly specifically in fruits, suggesting a role in maturation processes. *PaPHYA* showed high expression in seeds and leaves, while *PaPHYBa* exhibited negligible activity across tissues. Among HPs, *PaHP1* maintains stable and high expression levels across all tissues, with particularly remarkable expression in leaves. While others showed tissue-restricted patterns, *PaPHP1* significantly decreased in seeds and roots, *PaHP5* was undetectable in leaves and flowers, *PaHP7* nearly absent in roots and stems but active in leaves. RRs exhibited the most diverse expression profiles. While most RR genes were highly expressed in roots and leaves, several exhibited distinct tissue-specific patterns: *PaRR1* displayed extremely high expression in seeds; *PaRR2* was uniquely enriched in fruits and flowers, suggesting its potential role in reproductive development. Taken together, these expression patterns highlight the functional diversification of TCS members during distinct developmental stages.

### 3.5 Expression profiles of petunia TCS genes in response to exogenous plant growth regulators

**Figure 7:**
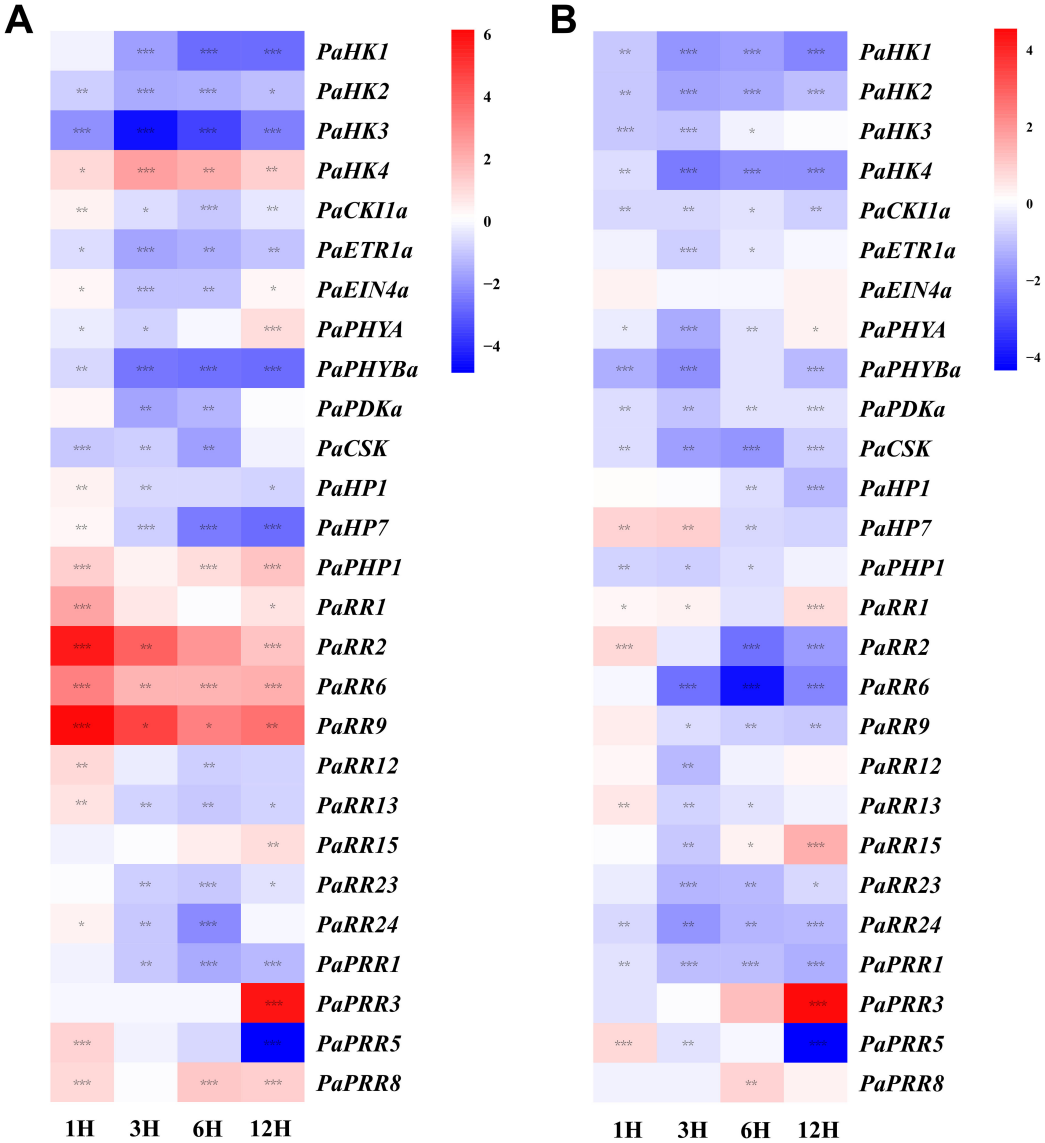
Heat map representation for the response patterns to exogenous tZ **(A)** and ABA **(B)** of TCS genes in petunia. Gene expression levels are presented using fold-change values transformed to Log_2_ format compared with control (0 h, value=0). The Log_2_ (fold-change values) and the color scale are shown at the right of heat map. Blue, white, and red represent low, medium, and strong expression, respectively. Asterisks denote statistical significance: *p < 0.05; **p < 0.01; ***p < 0.001. Detailed statistical analyses are provided in **Supplementary Figure S6**.

Transcriptional profiling of 27 petunia TCS genes (detected in leaves) revealed distinct regulatory dynamics under exogenous tZ (**Figure 7A**) and ABA (**Figure 7B**) treatments. Since *PaETR2b*, *PaHP5*, and *PaRR30* were not detected in leaves under these treatments, they were not further analyzed. Gene expression changes with |log_2_FC| > 1 and *p* < 0.05 were considered statistically significant.

It’s obvious that all detected type-A RRs (*PaRR1*, *PaRR2*, *PaRR6*, and *PaRR9*) were rapidly induced, particularly at 1 h after exogenous tZ treatment. Specifically, the mRNA level of *PaRR9* increased by nearly 72-folds at 1 h compared with the control (0 h), which is consistent with their conserved role as primary cytokinin-responsive genes across species such as *Arabidopsis*, tomato, and Chinese cabbage. In contrast, type-B RRs exhibited stable mRNA levels with no significantly upregulation or downregulation. Additionally, HK family genes displayed divergent responses: *PaHK4* was upregulated by nearly 5.6-fold at 3 h, while *PaHK1*–*PaHK3* were suppressed. Among HPs, *PaHP1* and *PaHP7* exhibited transient upregulation at 1 h but declined thereafter, with *PaHP7* being strongly suppressed at 6–12 h. Conversely, *PaPHP1* remained at a relatively high level (up to nearly 3-fold at 12h) after the treatment. Under ABA treatment, nearly all HKs were downregulated. Among HPs, *PaHP7* exhibited transient induction during the early stages (1–3 h), followed by a decline to 65% of the control at 6 h. Notably, *PaPHP1* exhibited distinct expression patterns under tZ and ABA treatments: its transcript levels remained at around two-fold of the control at 1 and 6 h under tZ treatment, while declining to 60–70% of the control at 1–6 h under ABA treatment. Furthermore, most petunia RRs were transcriptionally suppressed by ABA. The downregulation of type-A RRs (*PaRR1*, *PaRR2*, *PaRR6*, *PaRR9*), which were induced by cytokinin, reinforces the functional antagonism between ABA and cytokinin signaling pathways.

### 3.6 Expression profiles of petunia TCS genes in response to abiotic stresses

**Figure 8:**
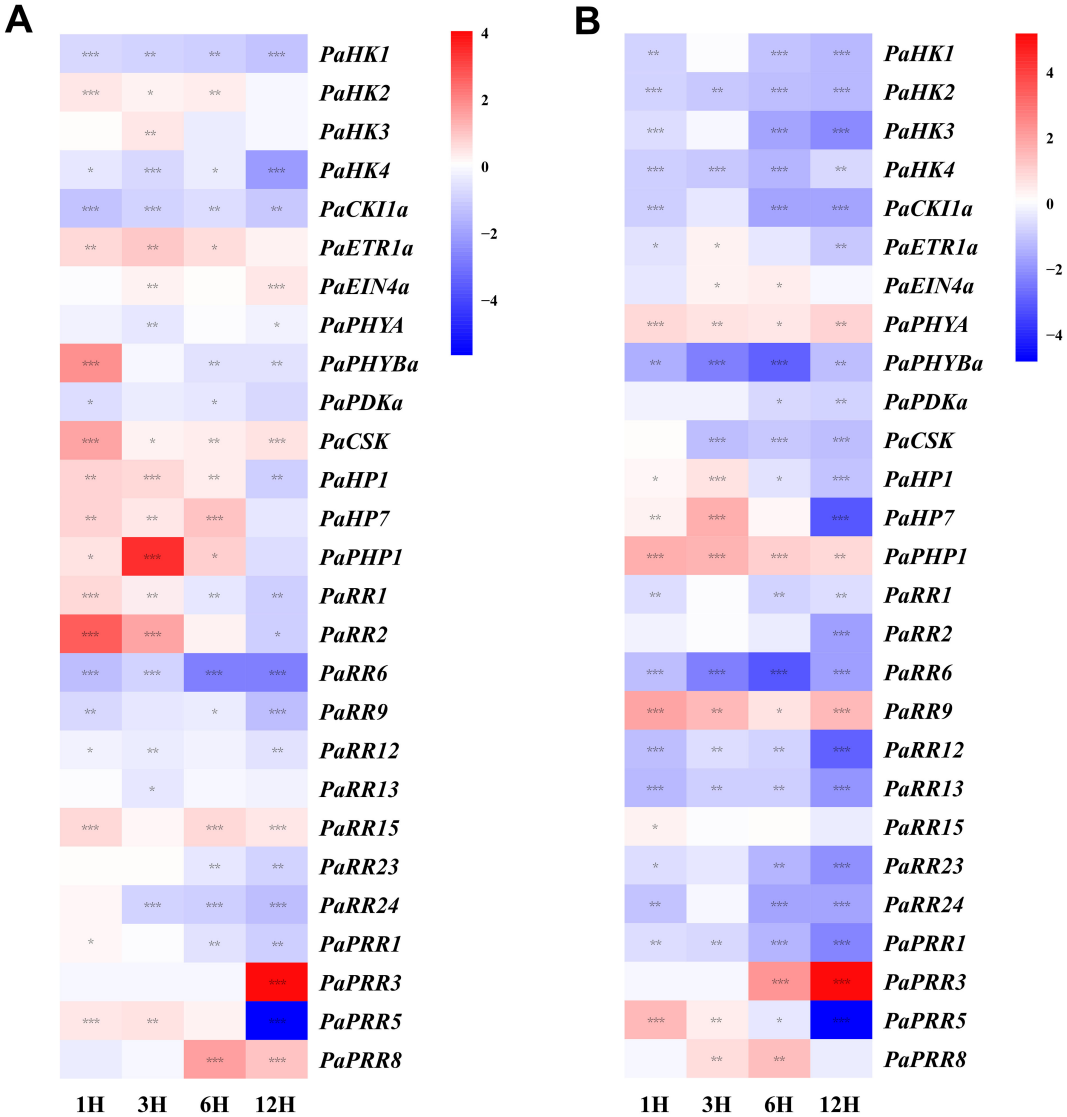
Heat map representation for the response patterns to drought treatment **(A)** and salt treatment **(B)** of TCS genes in petunia. Gene expression levels are presented using fold-change values transformed to Log_2_ format compared with control (0 h, value=0). The Log_2_ (fold-change values) and the color scale are shown at the right of heat map. Blue, white, and red represent low, medium, and strong expression, respectively. Asterisks denote statistical significance: *p < 0.05; **p < 0.01; ***p < 0.001. Detailed statistical analyses are provided in **Supplementary Figure S6**.

Drought and high salinity are two of the most critical stress factors affecting plant growth. Under drought treatment (**Figure 8A**), the expression of *PaHK1*, *PaHK4*, *PaCKI1a*, and *PaPDKa* was downregulated throughout the time course. By contrast, *PaPHYBa* and *PaCSK* were rapidly induced at 1h, while *PaHK2* and *PaETR1a* showed upregulation during the 1–6 h. Among HP genes, *PaHP1*, *PaHP7*, and *PaPHP1* showed increased expression during the 1–6 h; notably, *PaPHP1* transcript levels reached nearly 11-fold at 3h, though all three declined below the control level by 12 h. Within the RR family, most members (*PaRR6*, *PaRR9*, *PaRR12*, *PaRR23*, and *PaRR24*) were transcriptionally repressed. In contrast, *PaRR1* and *PaRR2* displayed transient upregulation, with *PaRR2* expression increasing approximately 6.5-fold at 1 h. Under salt treatment (**Figure 8B**), HK family members were generally downregulated, exhibiting an overall inhibitory trend. Specifically, *PaHK1–4* and *PaCKI1a* were significantly downregulated at 3 h, while *PaPHYA* showed sustained upregulation, contrasting with the significant downregulation of *PaPHYBa*. The response patterns of HP genes to drought and salt stress were similar, with *PaPHP1* maintaining consistently high expression levels (2- to 3.7-fold) throughout the treatment. Notably, within the RR family, only *PaRR9* exhibited a strong positive response to salt stress, while the majority of RR members were significantly transcriptionally repressed. *PaRR2* displayed a divergent transcriptional response to drought and salt stresses, with significant induction observed only under drought treatment within the first 1–3 h. In contrast, *PaRR9* was suppressed under drought yet induced by salt stress. Furthermore, under treatments of exogenous hormones and abiotic stresses, the clock-associated genes *PaPRR3* and *PaPRR5* exhibit pronounced circadian rhythms, indicating their functional role in circadian regulation.

## 4 Discussion

In this study, we identified 74 TCS genes in petunia, comprising 24 HK(L)s, 10 HPs, and 40 RRs. A comparative analysis of identified plant species reveals considerable variation in TCS gene numbers (**Supplementary Table S1**). *Brassica napus* harbors the largest TCS gene family (182 genes) among the plants studied, primarily due to segmental duplications during the process of polyploidization (Liu et al., 2023). In contrast, *Sorghum bicolor* (37 genes) and *Physcomitrella patens* (39 genes) contain fewer TCS members than other plants, potentially reflecting their small genome size and relatively few duplication events during their evolution (Ishida et al., 2010; Zameer et al., 2021). Similarly, the absence of whole-genome duplication events contributes to the small TCS family size in cucumber and watermelon (He et al., 2016b). The evolutionary expansion of the TCS family in *Arabidopsis*, Chinese cabbage (Liu et al., 2014), and soybean (Mochida et al., 2010) is driven mainly by segmental duplication, whereas both segmental and tandem duplications have contributed to its expansion in tomato (He et al., 2016a). Furthermore, the absence of type-C RR genes in certain species further underscores the evolutionary diversification of TCS signaling system.

Based on phylogenetic and functional analyses, PaHK(L)s constitute a functionally specialized and evolutionarily diversified gene family though the gene number is not large. In petunia, the number of divergent HKLs exceeds that of canonical HKs. These HKLs share sequence similarities with typical HKs, but lack HK activity. Instead, they have acquired serine/threonine kinase activity, and/or functionally integrate with serine/threonine signaling systems (Thelen et al., 2000; Moussatche and Klee, 2004; Shin et al., 2016). Interestingly, despite the loss of histidine-aspartate kinase activity, these HKLs may still utilize structural features derived from two-component signaling elements to facilitate signal transduction, such as ethylene receptors (Bisson and Groth, 2010) or interaction between phytochromes and type-A response regulators (Sweere et al., 2001). This functional divergence reflects multiple evolutionary adaptations to meet the complex signaling requirements of multicellular eukaryotes. We also identified three *CKI1* homologs in petunia, whereas both *Arabidopsis* and tomato possess only one. In *Arabidopsis*, *CKI1* acts upstream of *AHP2*, *AHP3*, and *AHP5* to activate the TCS pathway in parallel with cytokinin receptors (Liu et al., 2017) and can also regulate female gametophyte development and vegetative growth (Hejátko et al., 2003, 2009; Deng et al., 2010). The *CKI1* ortholog in rice is involved in female gametophyte development (Ito and Kurata, 2006), while mutation of *CaCKI1* causes seedless fruits in chili pepper (Maki et al., 2023). *PaCKI1a* is highly expressed in seeds and fruits, suggesting a potential role in reproductive development as well. However, whether the three *CKI1* homologs in petunia have undergone functional divergence remains to be investigated. Phylogenetic analysis of HPs clearly separates monocot and dicot lineages into distinct clades, indicating high conservation within each group. Petunia possesses three pseudo-HPs, whereas *Arabidopsis* and tomato possess only one and two, respectively. Given that pseudo-HPs function as competitive inhibitors of phosphoryl transfer from HKs to authentic HPs, this difference in gene copy number suggests an increased regulatory complexity of phosphotransfer processes in petunia. PaRRs could be divided into four subfamilies. It has been reported that type-C RRs are the oldest subgroup and type-A RRs are the youngest in the evolution of the RRs. Type-A and type-C RRs were clustered into different branches and their genetic distance was not closely related although they shared a similar structure. Type-B PRRs are more closely related to type-B RRs than to clock PRRs, likely to be evolved from the type-B RRs. All the components of the TCS pathway might have been recruited at different time points during evolution to give rise to the signaling system found in land plants (Pils and Heyl, 2009). We next investigated the expression patterns of TCS genes to gain insights into their potential biological functions. Expression analysis revealed tissue-specific expression of TCS genes in petunia, with the highest transcript abundance in leaves and roots. This is consistent with the fact that roots are regarded as the most crucial primary organ for responding to drought and high-salinity stresses, and cytokinin are mainly synthesized in the roots (Sánchez-Blanco et al., 2014; Wu et al., 2021). It is also worth mentioning that PaHPs function as phosphohistidine intermediates and exhibit tissue-specific expression, which enables HP members to precisely regulate signal transduction at distinct sites. The cytokinin signaling pathway is a typical representative of the TCS signaling system, where cytokinin treatment rapidly induces type-A RR genes in petunia, consistent with their function as primary cytokinin-responsive factors across species such as *Arabidopsis* (Kiba et al., 1999), tomato (He et al., 2016a), Chinese cabbage (Liu et al., 2014), and most other plants. However, the expression of type-B RRs remains relatively stable, likely due to their post-transcriptional regulation rather than transcriptional activation by cytokinin (Xie et al., 2018). In *Arabidopsis*, type-B ARRs target negative regulators of hormone signaling (e.g., type-A *ARRs*, *Aux*/*IAA*, and *EBF*s) when the level of cytokinin increases, enabling rapid rebalancing of hormonal responses. Especially, Type-B ARRs, functioning as transcription factors, directly bind to promoters of type-A *ARRs*, activating their transcription (Hwang and Sheen, 2001; To et al., 2004; Lee et al., 2008). This establishes a negative feedback loop, but the mechanisms are unknown. It has been proposed that upon induction, type-A ARRs suppress cytokinin signaling via competitive binding to phosphoryl groups or phosphospecific interactions with downstream targets (To et al., 2007; Kim, 2008). Furthermore, extensive crosstalk exists between cytokinin and ABA (Ha et al., 2012). In petunia, most TCS genes show the downregulation of transcript levels under ABA treatment, which consistent with expression patterns in Chinese cabbage but opposite to the induction observed in tomato.

Accumulating evidence demonstrates that TCS serves as a central signaling network involved in responses to various abiotic stresses (Tran et al., 2007; Jeon et al., 2010; Pham et al., 2012; Wen et al., 2015). However, it exhibits both conserved and divergent stress-response strategies across plant species. In petunia, drought treatment transiently upregulates *PaHP1*, *PaHP7*, *PaPHP1*, *PaRR1*, and *PaRR2* while suppressing most RRs, paralleling with the broad repression pattern in Chinese cabbage (Liu et al., 2014). This contrasts with sorghum (Zameer et al., 2021), where drought induces RRs, and tomato (He et al., 2016a), where most HPs and PRRs are upregulated under drought stress. In *Arabidopsis*, drought downregulates AHPs (*AHP2*, *AHP3*, *AHP5*) (Nishiyama et al., 2013) and type-B ARRs (*ARR1*, *ARR10*, *ARR12*) (Nguyen et al., 2016), while strongly inducing type-A *ARRs* (*ARR5*, *ARR7*, *ARR15*). By contrast, rice exhibits suppression of nearly all type-A RRs under drought (Jain et al., 2008). Salt stress similarly represses most TCS genes in petunia, except *PaPHYA*, *PaPHP1*, *PaRR9*, and *PaPRR8*, which show positive responses. This pattern aligns with the repression observed in tomato (He et al., 2016a), soybean (Mochida et al., 2010), and Chinese cabbage (Liu et al., 2014). Functional studies in *Arabidopsis* reveal that *AHK1* positively regulates drought and ABA signaling (Tran et al., 2007), whereas its petunia homolog *PaHK1* is downregulated under these treatments. Additionally, comparison of TCS gene expression profiles between petunia, *Arabidopsis*, tomato (He et al., 2016a), and *Brassica napus* (Liu et al., 2023) reveals that even homologous TCS genes exhibit divergent stress responses, further highlighting functional diversification across species. While the expression profiling provides initial insights, the functional roles of petunia TCS genes in abiotic stress adaptation remain to be fully elucidated. Notably, *PaPHP1* is implicated in both drought and salt stress responses, *PaRR2* appears specific to drought responses, and *PaRR9* to high-salinity stress responses. Furthermore, approximately 18% of the investigated genes, including *PaETR1a*, *PaEIN4a*, *PaPHYA*, *PaRR1*, *PaRR15*, exhibited no significant response (|log_2_FC| < 1) to the applied stress treatments. It is speculated that some of these genes may functions in other tissues or under specific conditions. For instance, *Arabidopsis ETR2* stimulates seed germination under salt stress (Wilson et al., 2014), and the responses of BnaTCSs to salt stress were different in leaves, roots, and seeds (Liu et al., 2023).To further elucidate the regulatory mechanisms underlying the observed expression patterns, we analyzed CREs in the promoter regions of petunia TCS genes. Our analysis showed that nearly all genes contained motifs theoretically responsive to ABA, drought, and salt stress. Among these, 63 out of the total 74 genes contained ABRE in their promoter regions, 24 of which showed transcriptional repression. Similarly, 40 genes contained MBS in their promoter regions, with 16 genes transcriptionally repressed. The inconsistent results between expression profiles and promoter analyses were also observed in tomato (He et al., 2016a), soybean (Mochida et al., 2010), and Chinese cabbage (Liu et al., 2014). It is speculated that the sequences of some CREs might be structurally correct without providing a practical regulatory function. Such discrepancies could also stem from complex interactions within hormonal and stress regulatory networks and stress-repressive genes among the TCS genes. Moreover, the accurate identification of CREs is highly dependent on the quality and completeness of the genome assembly, which represents another potential source of variation.

In summary, while this study provides a foundational characterization of TCS genes in petunia, several limitations merit consideration. Firstly, the current scaffold-level genome assembly of *P. axillaris* may affect the accuracy of gene annotations. Future studies utilizing improved genome assemblies will help verify the genomic organization of the identified TCS genes. Secondly, our functional inferences rely primarily on bioinformatic and expression analyses, while direct validation of key genes, particularly cytokinin receptors and stress-responsive candidates including *PaPHP1*, *PaRR2*, and *PaRR9*, through gene overexpression or CRISPR-based gene editing remains to be conducted. Such validation would help clarify the roles of the TCS genes in mediating petunia growth and stress adaptation. Furthermore, although protein–protein interactions between TCS components (AHK–AHP and AHP–ARR) have been characterized in *Arabidopsis* (Huo et al., 2020), they remain uncharacterized in petunia. Future work could employ yeast two-hybrid assays to systematically map HK–HP–RR interactomes in petunia. Overall, clarifying the functional specialization among TCS members will be essential for comprehensively understanding how this signaling system regulates plant development and environmental stress responses.

## 5 Conclusion

This study provides the first comprehensive genome-wide identification and characterization of TCS genes in petunia, providing critical insights into TCS-mediated signaling mechanisms in this key ornamental species. Analysis of CREs and expression profiling under drought, salt, and hormone treatments demonstrated that TCS genes are likely involved in integrating diverse environmental and hormonal signals. Notably, several TCS members, including *PaPHP1*, *PaRR2*, and *PaRR9*, exhibited pronounced stress-responsive expression, while cytokinin receptor genes have been documented to regulate floral development, particularly flower size. These genes represent promising candidates for in-depth functional studies. Further studies should employ genetic approaches, including gene overexpression, CRISPR-based gene editing, and protein-protein interaction assays, to fully elucidate the roles of these TCS genes. Overall, this study establishes a foundational framework for future research on TCS pathways, with the potential to inform strategies for improving ornamental traits (e.g., flower size and longevity) and stress tolerance in petunia through molecular breeding.

## Data Availability

The datasets presented in this study can be found in online repositories. The names of the repository/repositories and accession number(s) can be found in the article/**Supplementary Material**.

## Supplementary material

Supplementary Figure 1 Domain structures of PaHK(L)s.

Supplementary Figure 2 Amino acid sequence alignment of PaHK(L)s.

Supplementary Figure 3 Domain structures and amino acid sequence alignment of PaHPs.

Supplementary Figure 4 Domain structures of PaRRs.

Supplementary Figure 5 Amino acid sequence alignment of PaRRs.

Supplementary Figure 6 The expression patterns of petunia TCS genes in response to various hormone [tZ **(A)** and ABA **(B)**] and stresses [drought **(C)** and salt **(D)**].

Supplementary Table 1 Summary of the TCS gene numbers identified in plants.

Supplementary Table 2 Members of the TCS gene families identified in petunia genome.

Supplementary Table 3 Similarity of amino acid sequences of the HK(L), HP and RR proteins from *Arabidopsis* and petunia.

Supplementary Table 4 Summary of *cis*-regulatory elements in promoter region of petunia TCS genes.

Supplementary Table 5 Protein sequences of petunia HK(L)s, HPs and RRs.

Supplementary Table 6 Gene-specific primers used in qRT-PCR expression analysis of petunia TCS genes.
