# Correction: Genome-wide identification and functional characterization of the two-component system gene family in petunia reveals roles in hormone signaling and stress response

**DOI:** 10.3389/fpls.2026.1781669

**Published:** 2026-02-12

**Authors:** Binbin Dai, Juntao Huo, Linxia Zhang, Peishan Zou, Miaomiao Sun, Seping Dai, Guofeng Liu

**Affiliations:** Guangzhou Collaborative Innovation Center on Science-Tech of Ecology and Landscape, Guangzhou Institute of Forestry and Landscape Architecture, Guangzhou, China

**Keywords:** petunia, two-component system (TCS), cytokinin signaling, gene expression profiling, response regulator

There was a mistake in **Figures 1**–**8** as published. The resolution of the figures was insufficient for clear visualization of the presented data. The corrected **Figures 1**–**8** appears below.

**Figure 1:**
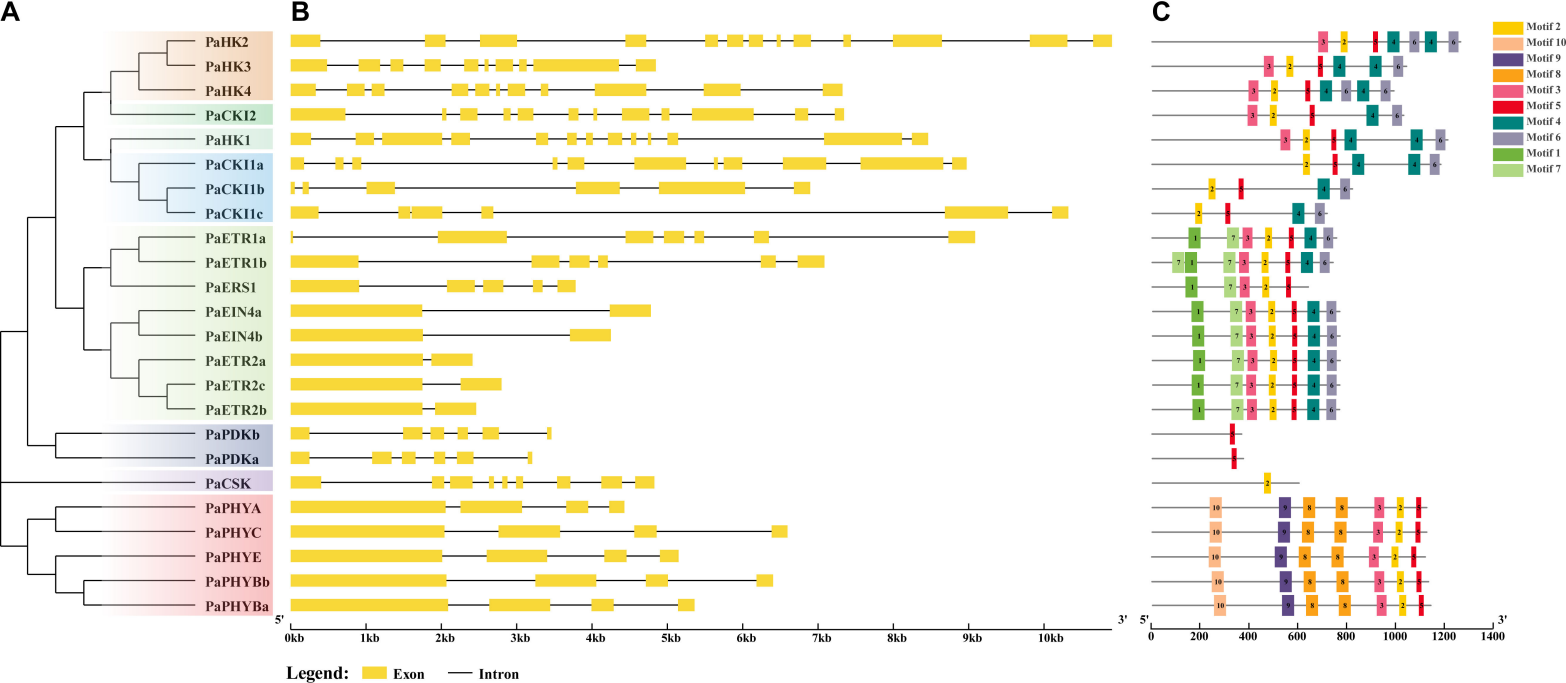
Phylogenetic relationships, gene structures, and conserved motifs of PaHK(L) genes. **(A)** Phylogenetic tree constructed based on the full-length sequences of 24 PaHK(L) proteins. **(B)** Exon–intron structure, analyzed with GSDS (yellow boxes: exons; black lines: introns). **(C)** Conserved motifs from PaHK(L) proteins are displayed in different colored boxes. The number below refers to the length of the protein.

**Figure 2:**
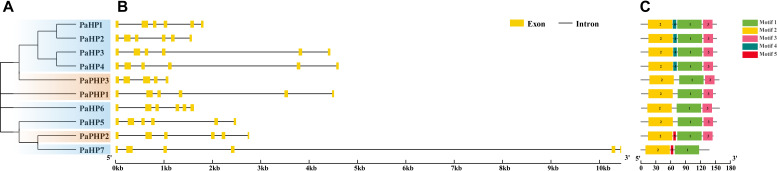
Phylogenetic relationships, gene structures, and conserved motifs of PaHP genes. **(A)** Phylogenetic tree constructed based on the full-length sequences of 10 PaHP proteins. **(B)** Exon–intron structure, analyzed with GSDS (yellow boxes: exons; black lines: introns). **(C)** Conserved motifs from PaHP proteins are displayed in different colored boxes. The number below refers to the length of the protein.

**Figure 3:**
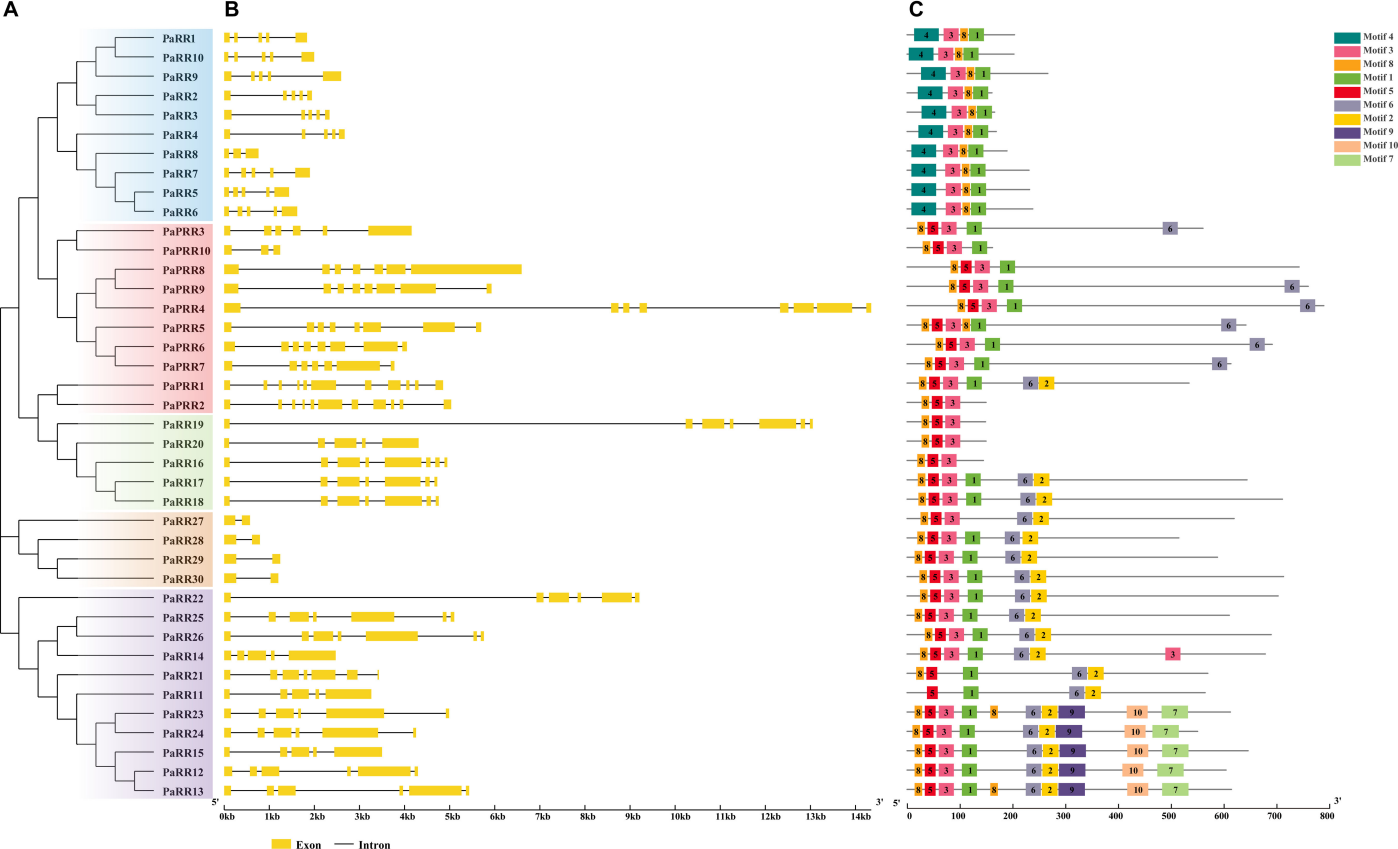
Phylogenetic relationships, gene structures, and conserved motifs of PaRR genes. **(A)** Phylogenetic tree constructed based on the full-length sequences of 40 PaRR proteins. **(B)** Exon–intron structure, analyzed with GSDS (yellow boxes: exons; black lines: introns). **(C)** Conserved motifs from PaRR proteins are displayed in different colored boxes. The number below refers to the length of the protein.

**Figure 4:**
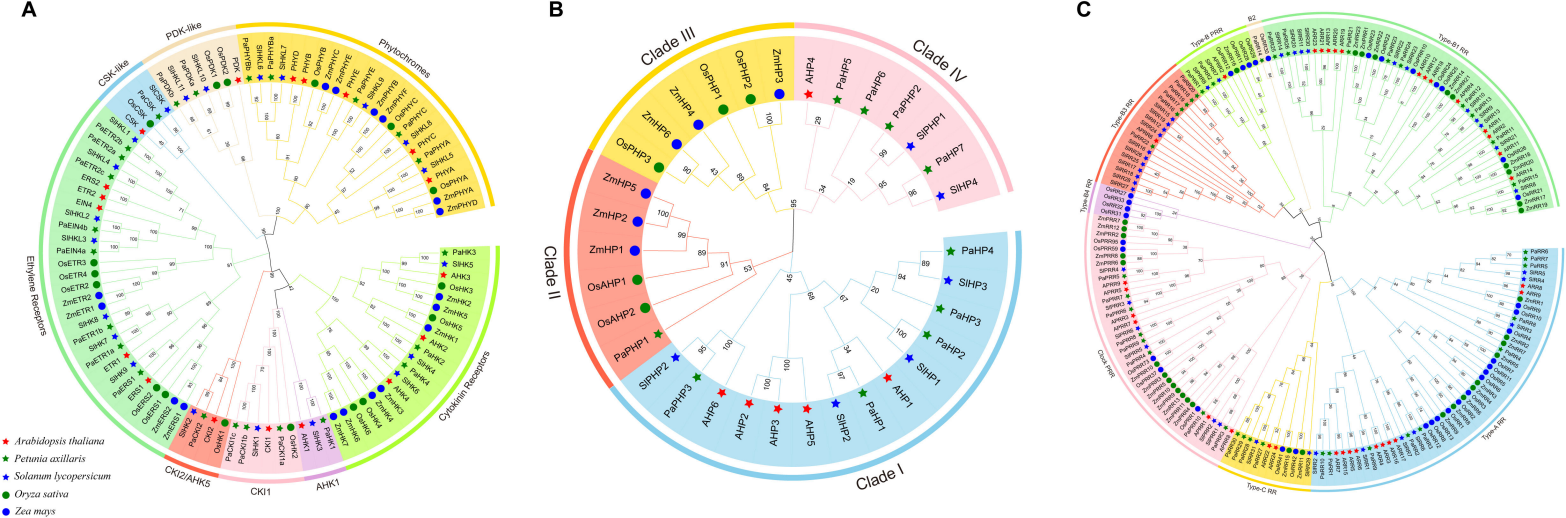
Phylogenetic relationships of HK(L)s **(A)**, HPs **(B)** and RRs **(C)** in *Arabidopsis*, rice, maize, tomato, and petunia. Different subfamilies are represented by different colors. Bootstrap values from 1000 replicates are shown at key nodes.

**Figure 5:**
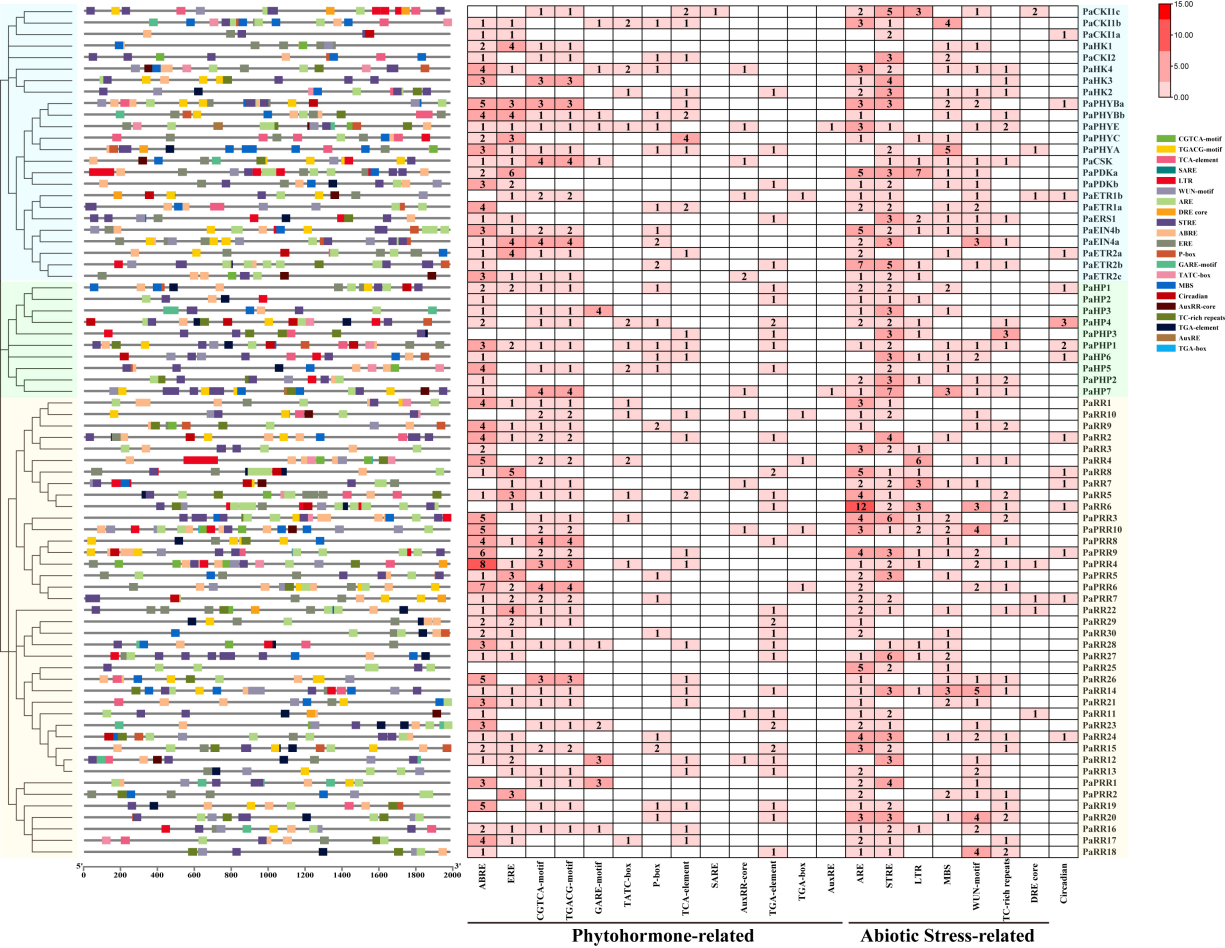
Distribution (left) and number (right) of CREs identified in putative promoter regions of TCS genes in petunia. The numbers in the heatmap represent the quantity of elements.

**Figure 6:**
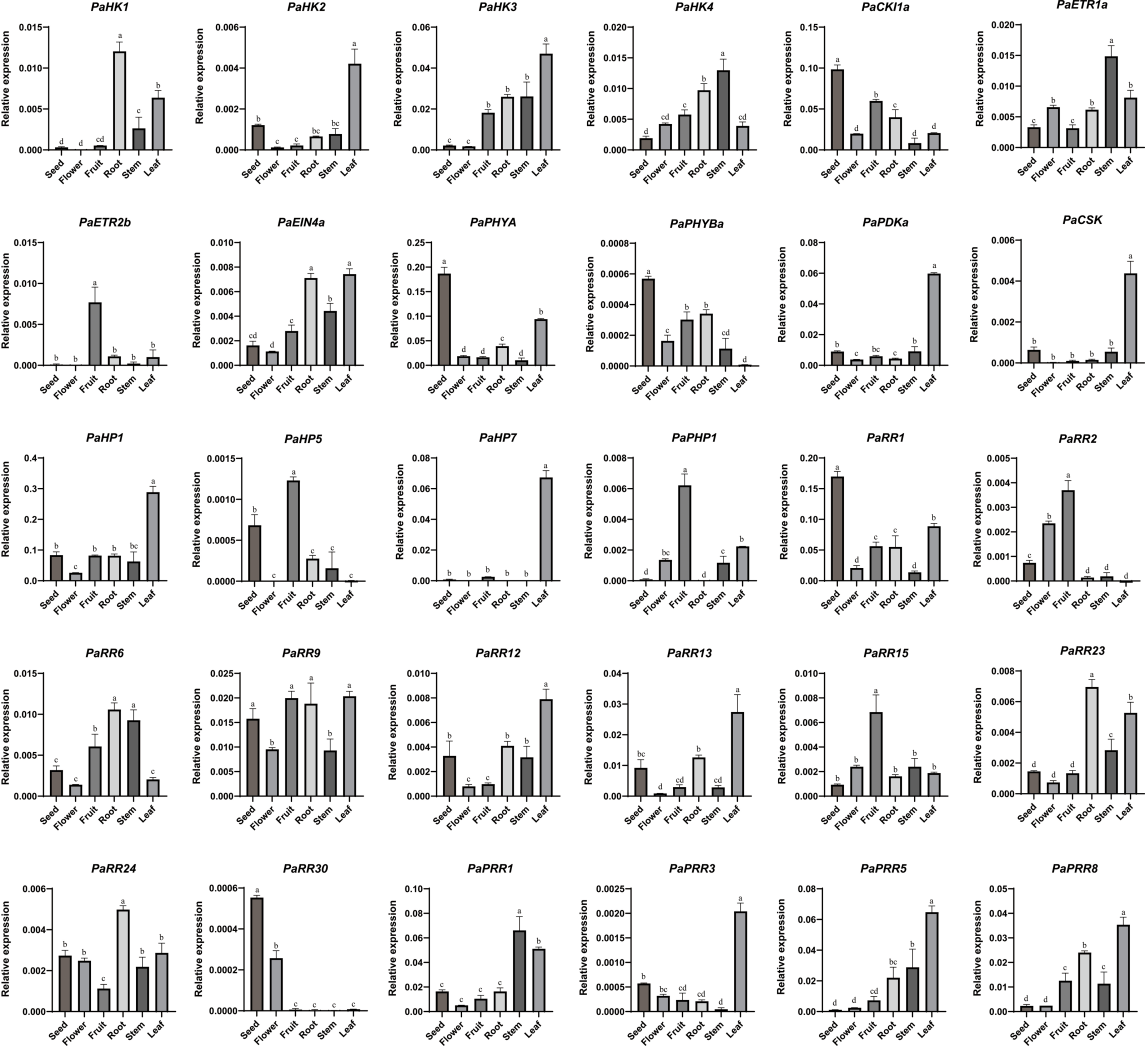
Organ-specific expression profiles of TCS genes in petunia. The relative expression level was normalized to the petunia *EF1α* gene and quantified using the 2^−ΔCT^ method. Data represent mean ± SD (standard deviation) values from three biological replicates per tissue. Significant differences (letters a-d above the bars) among tissues were analyzed by one-way ANOVA with Tukey’s *post-hoc* test (*p* < 0.05).

**Figure 7:**
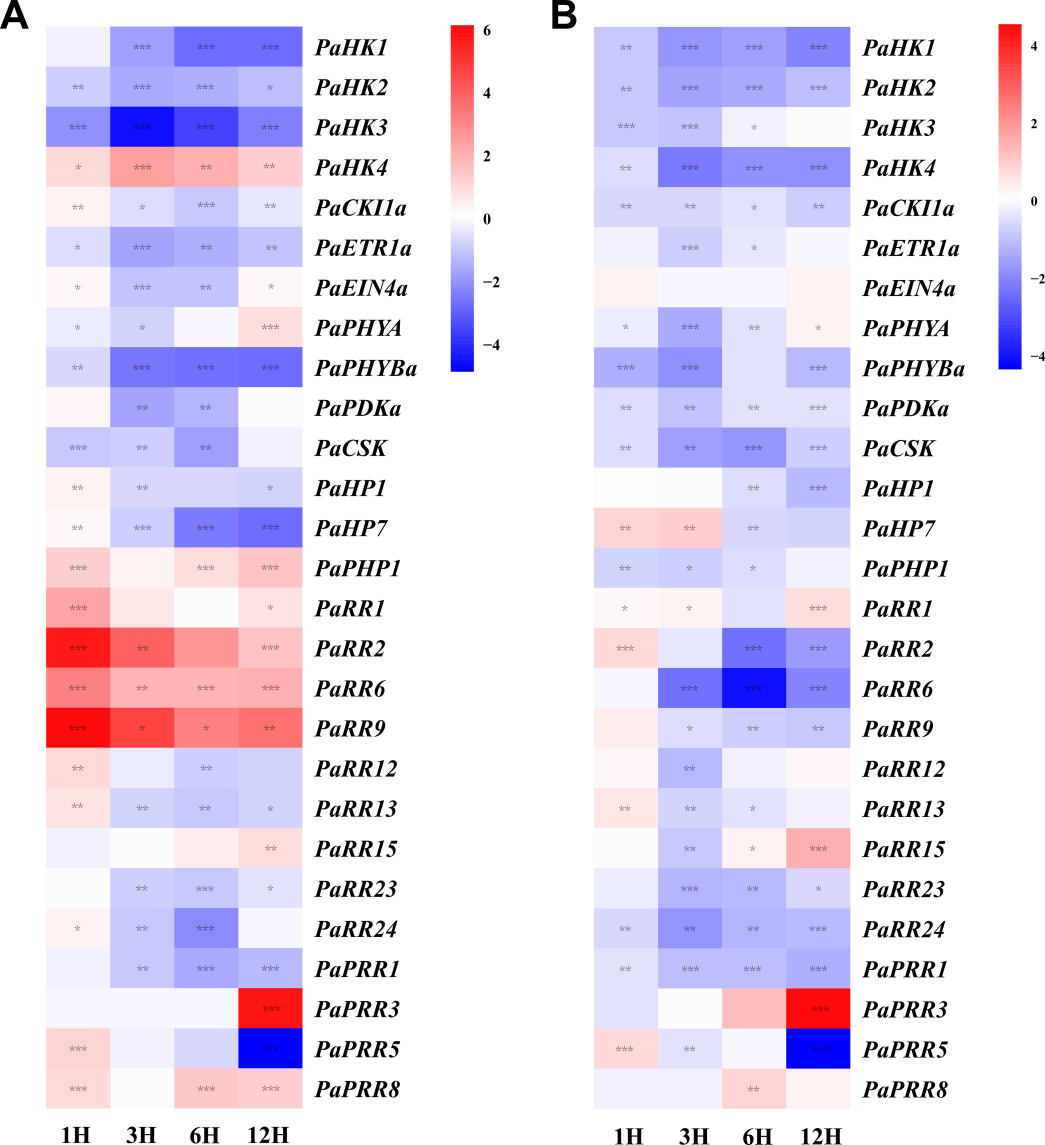
Heat map representation for the response patterns to exogenous tZ **(A)** and ABA **(B)** of TCS genes in petunia. Gene expression levels are presented using fold-change values transformed to Log_2_ format compared with control (0 h, value=0). The Log_2_ (fold-change values) and the color scale are shown at the right of heat map. Blue, white, and red represent low, medium, and strong expression, respectively. Asterisks denote statistical significance: *p < 0.05; **p < 0.01; ***p < 0.001. Detailed statistical analyses are provided in **Supplementary Figure S6**.

**Figure 8:**
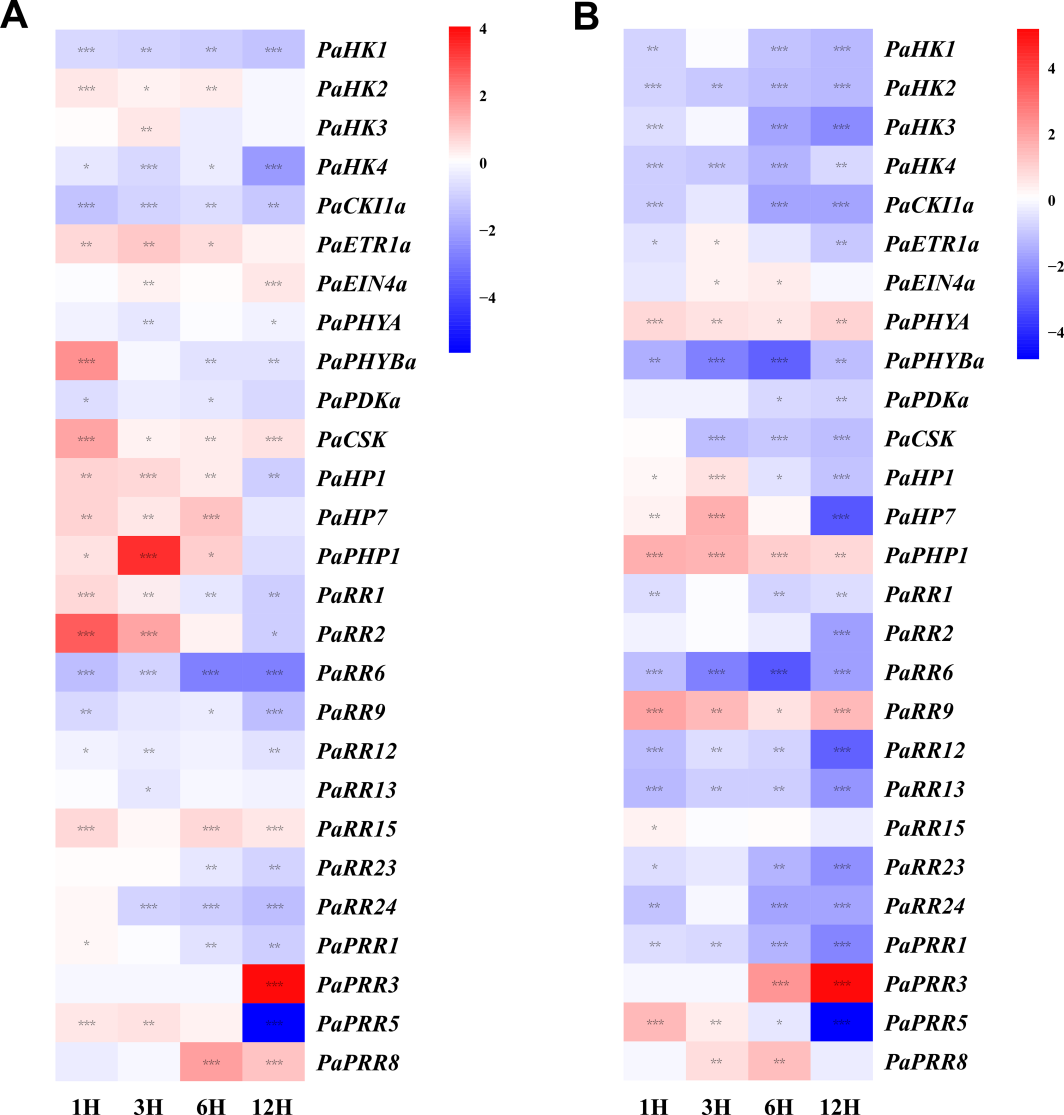
Heat map representation for the response patterns to drought treatment **(A)** and salt treatment **(B)** of TCS genes in petunia. Gene expression levels are presented using fold-change values transformed to Log_2_ format compared with control (0 h, value=0). The Log_2_ (fold-change values) and the color scale are shown at the right of heat map. Blue, white, and red represent low, medium, and strong expression, respectively. Asterisks denote statistical significance: *p < 0.05; **p < 0.01; ***p < 0.001. Detailed statistical analyses are provided in **Supplementary Figure S6**.

The original version of this article has been updated.

