# Supplementary material for: Genome-wide identification and functional characterization of the two-component system gene family in petunia reveals roles in hormone signaling and stress response
Source: Front Plant Sci. 2025 Dec 15;16:1721349. doi: 10.3389/fpls.2025.1721349 (PMC12745483; doi:10.3389/fpls.2025.1721349)
Supplement: Supplementary Figure 1 — Domain structures of PaHK(L)s. [file DataSheet1.zip › Supplementary Materials/Supplementary Figures.docx]

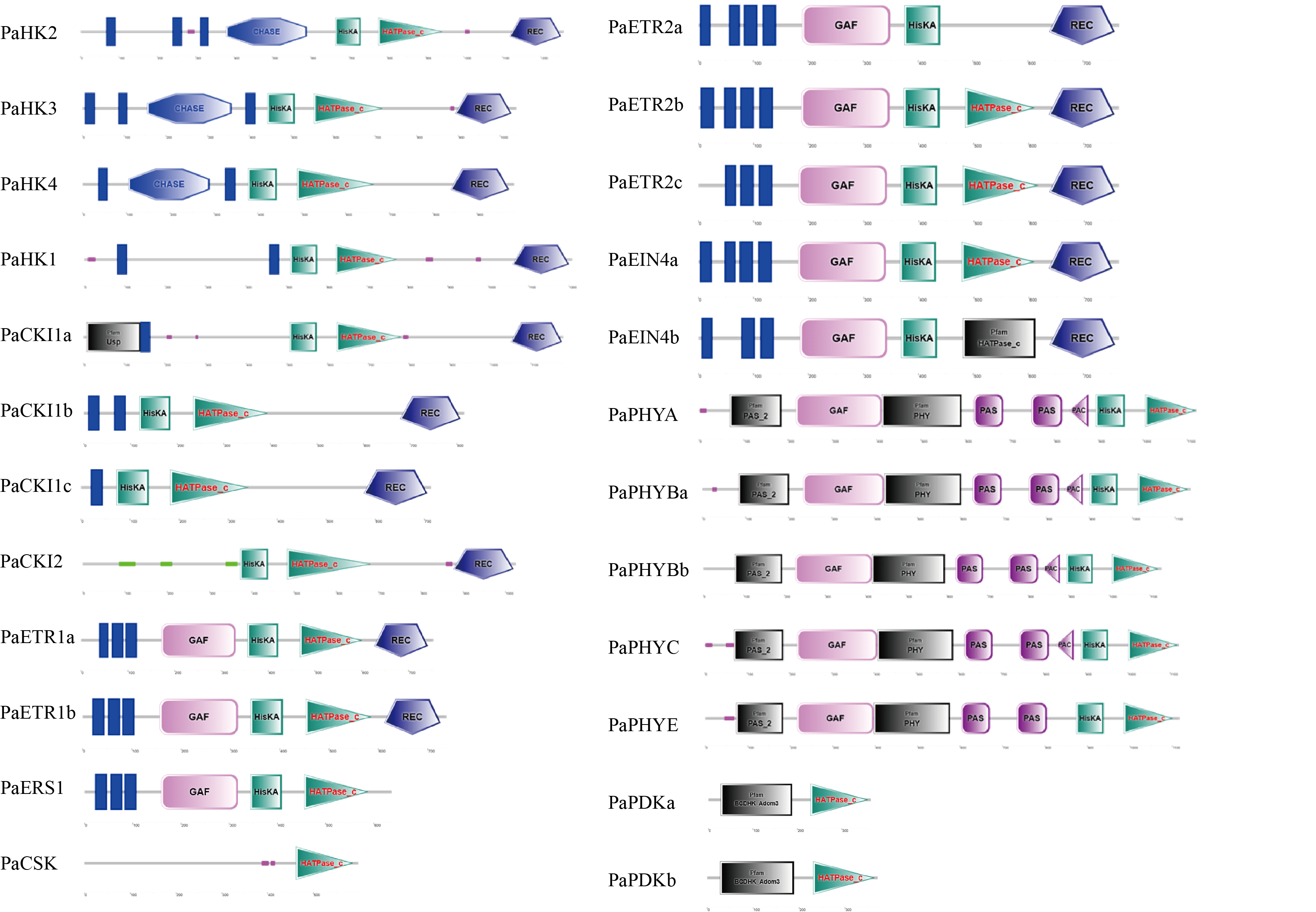


**Figure S1.** Domain structures of PaHK(L)s. Domain structures were analyzed by SMART online tool and drawn according to their original location and size. TM, transmembrane; HisKA, His Kinase A (phosphoacceptor) domain; HATPase, Histidine kinase-like ATPases; REC, receiver; CHASE, cyclases/histidine kinases associated sensing extracellular; GAF, cGMP phosphodiesterase/adenylyl cyclase/FhlA; PHY, phytochrome-specific GAF-related; PAS, Period/ARNT/Single-minded.


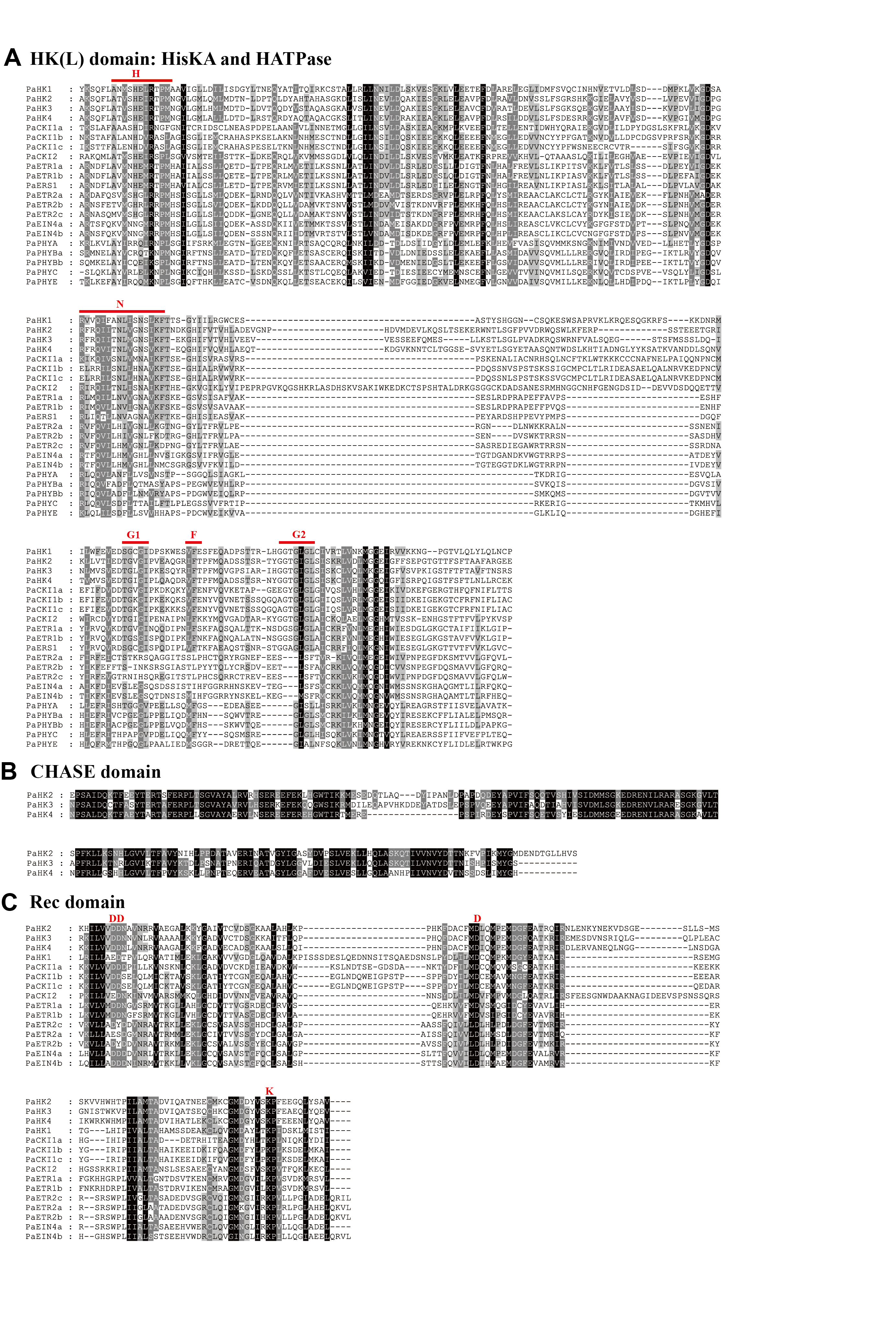


**Figure S2.** Amino acid sequence alignment of PaHK(L)s. (A) HK(L) (HisKA and HATPase); (B) Rec; (C) CHASE domains. The HK(L) domain was defined as the sequence extending from the phospho-acceptor domain through the ATPase domain. Black and gray backgrounds indicate percentage of amino acid similarity: black, at least 75%; darker gray, 50%; and lighter gray, 25%. Amino acid similarity groups are: D, N; E, Q; S, T; K, R; F, Y, and W; and L, I, V, and M.


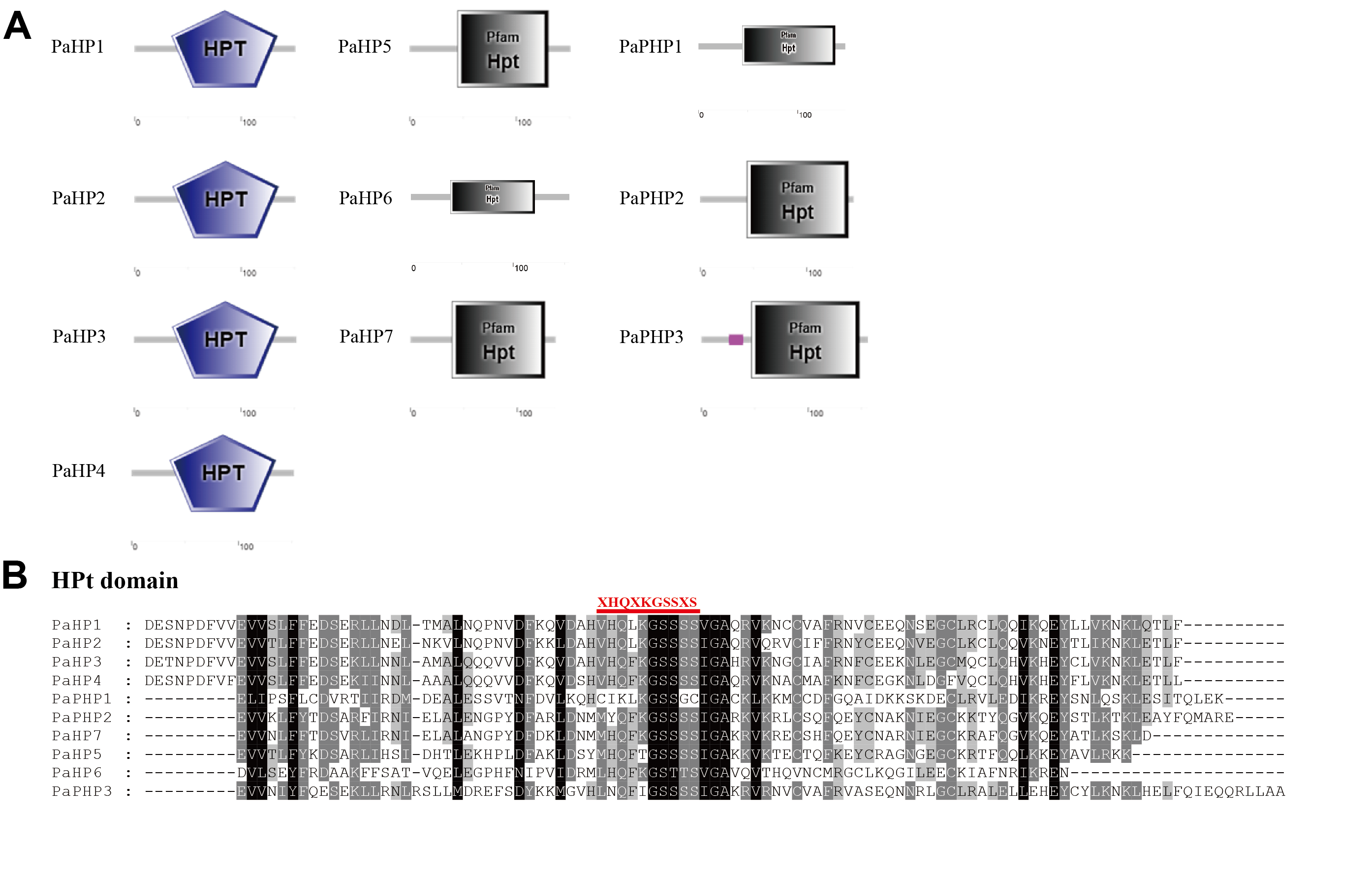
**Figure S3.** Domain structures and amino acid sequence alignment of PaHPs. (A) Domain structures were analyzed by SMART online tool and drawn according to their original location and size. (B) Amino acid sequence alignment of PaHPs. HPt, His-containing phosphotransfer.


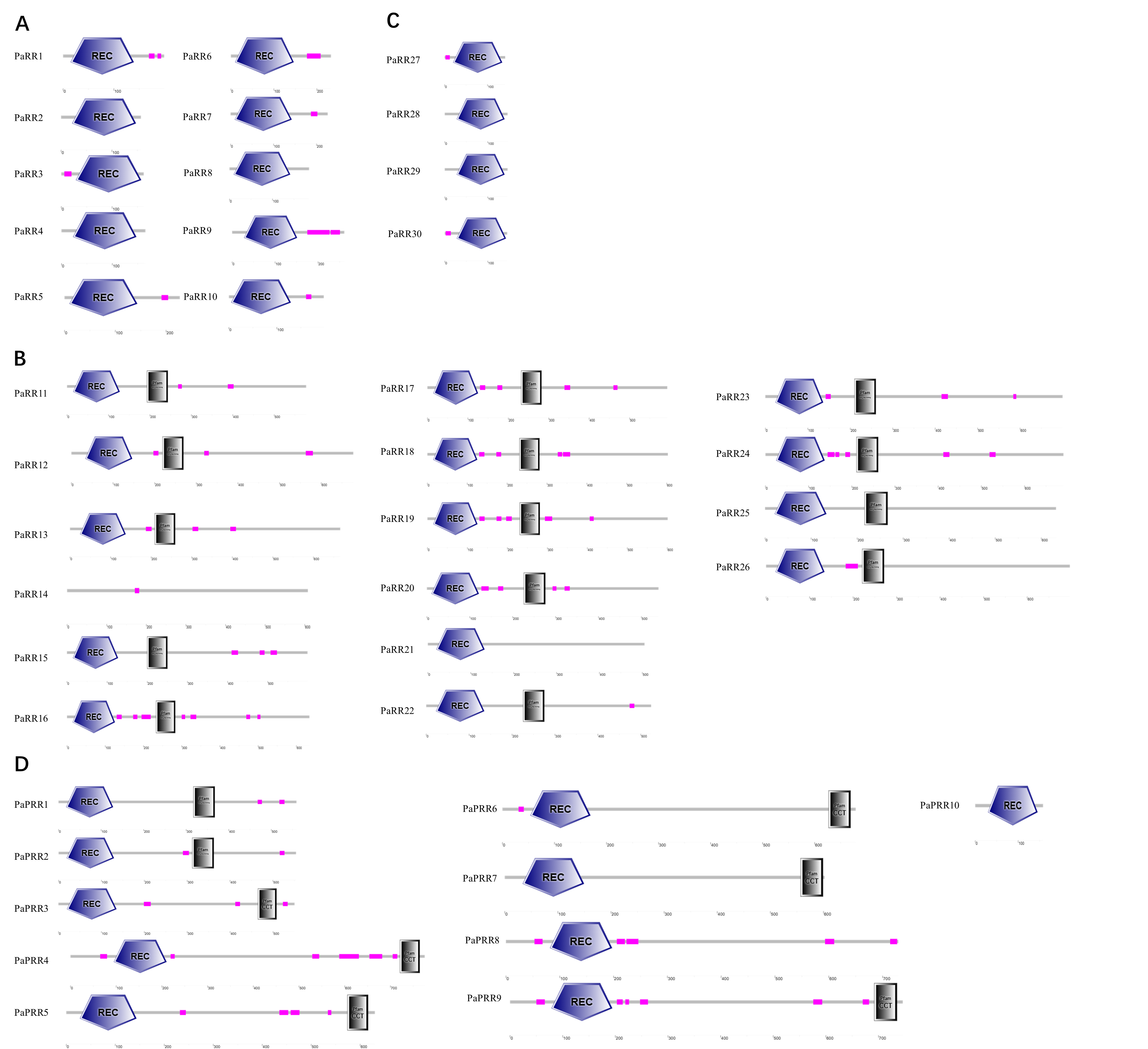


**Figure S4.** Domain structures of PaRRs. (A–D) represent the domain structures of type-A RR, type-B RR, type-C RR, and PRR, respectively. Rec, receiver; Myb, Myb DNA-binding; CCT, plant-specific CCT motif.


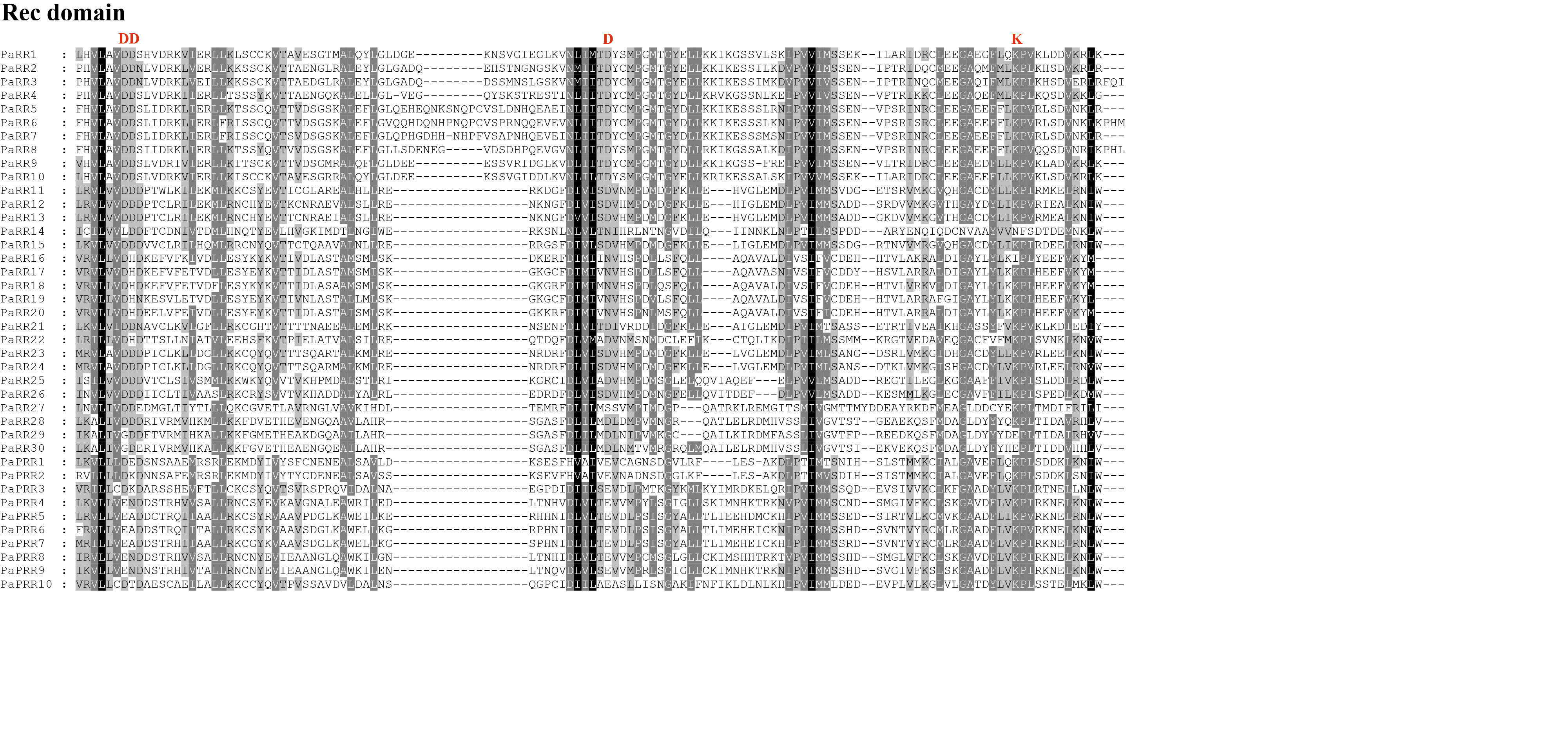


**Figure S5.** Amino acid sequence alignment of Rec domain from PaRRs.


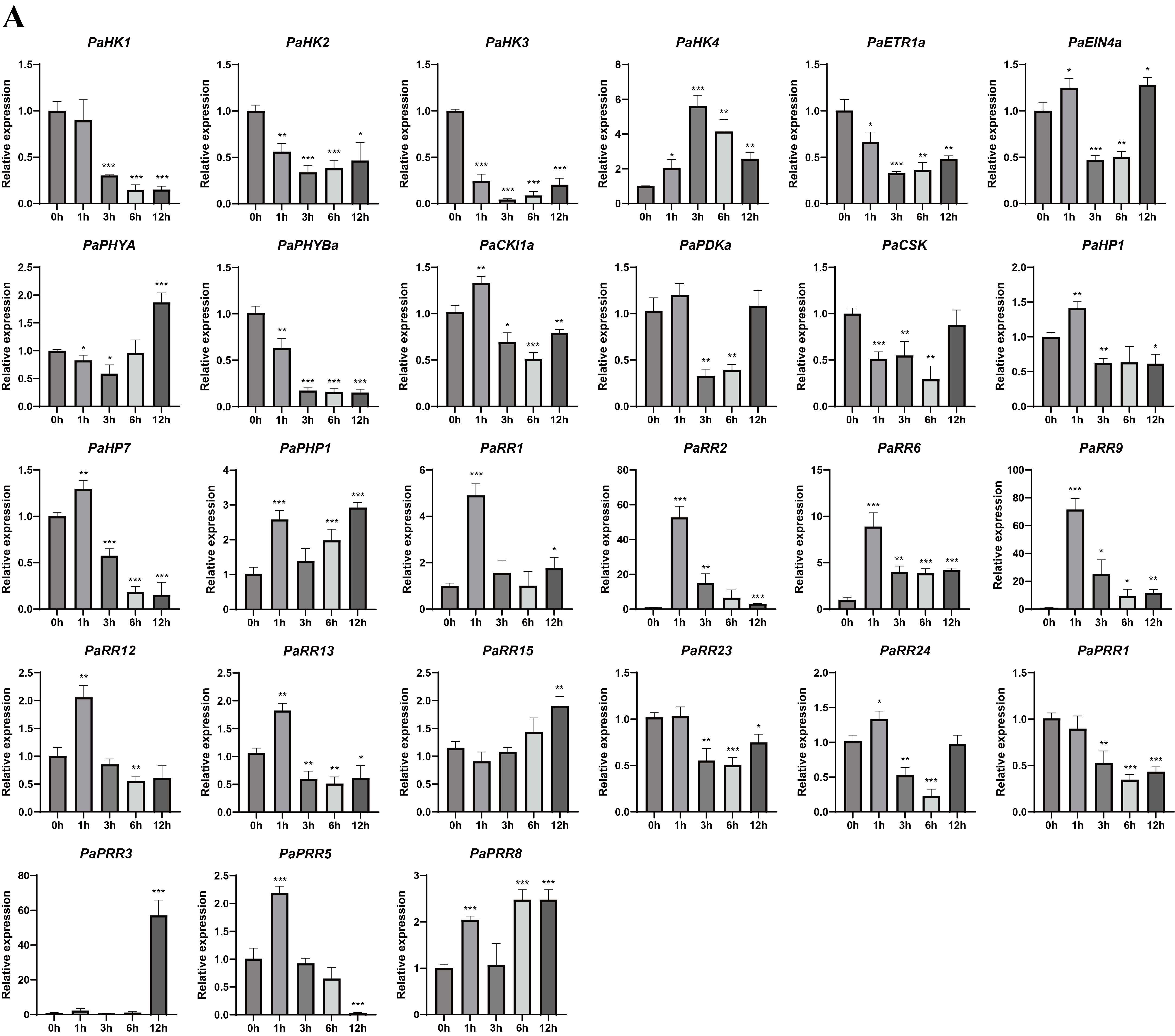


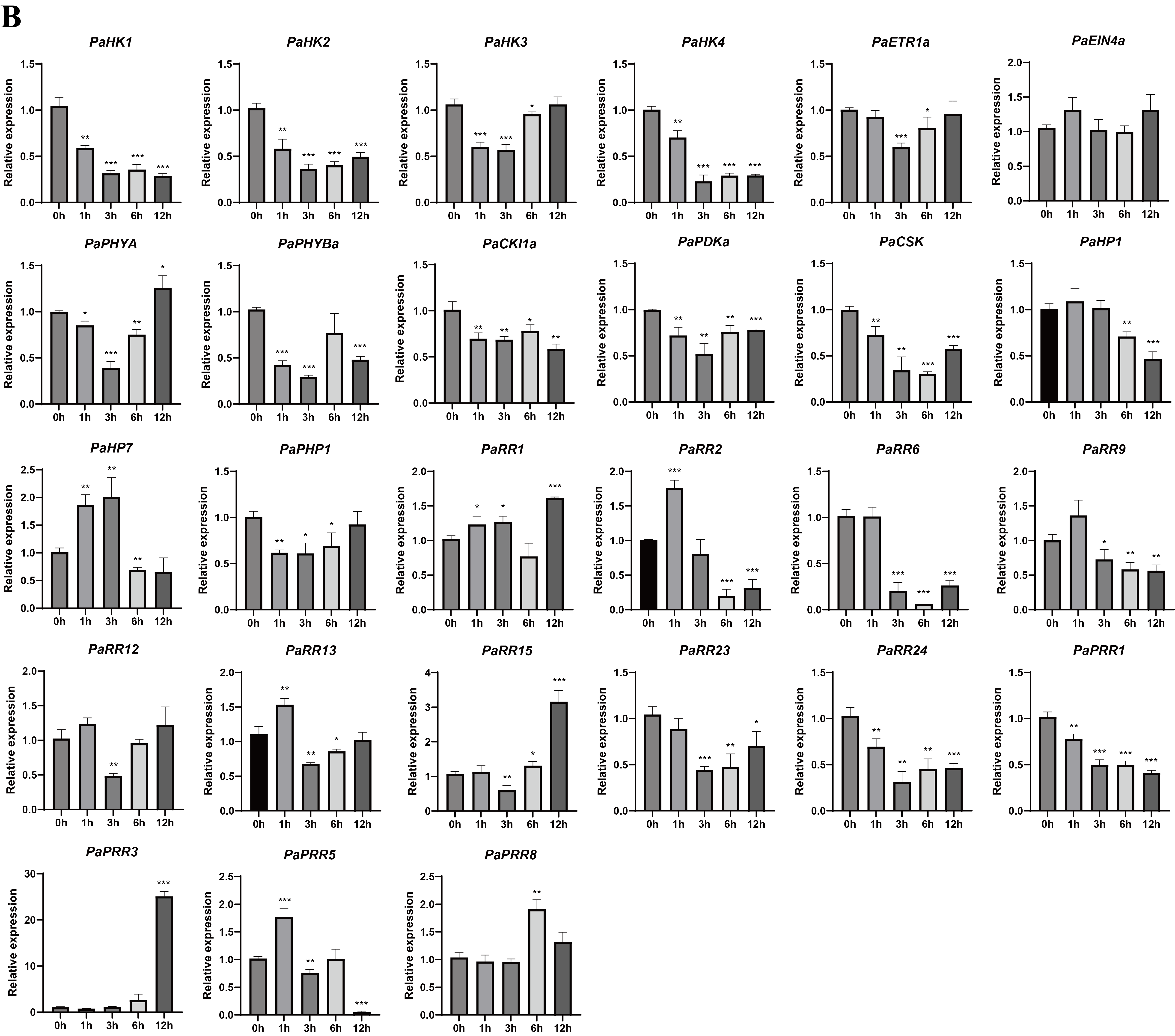


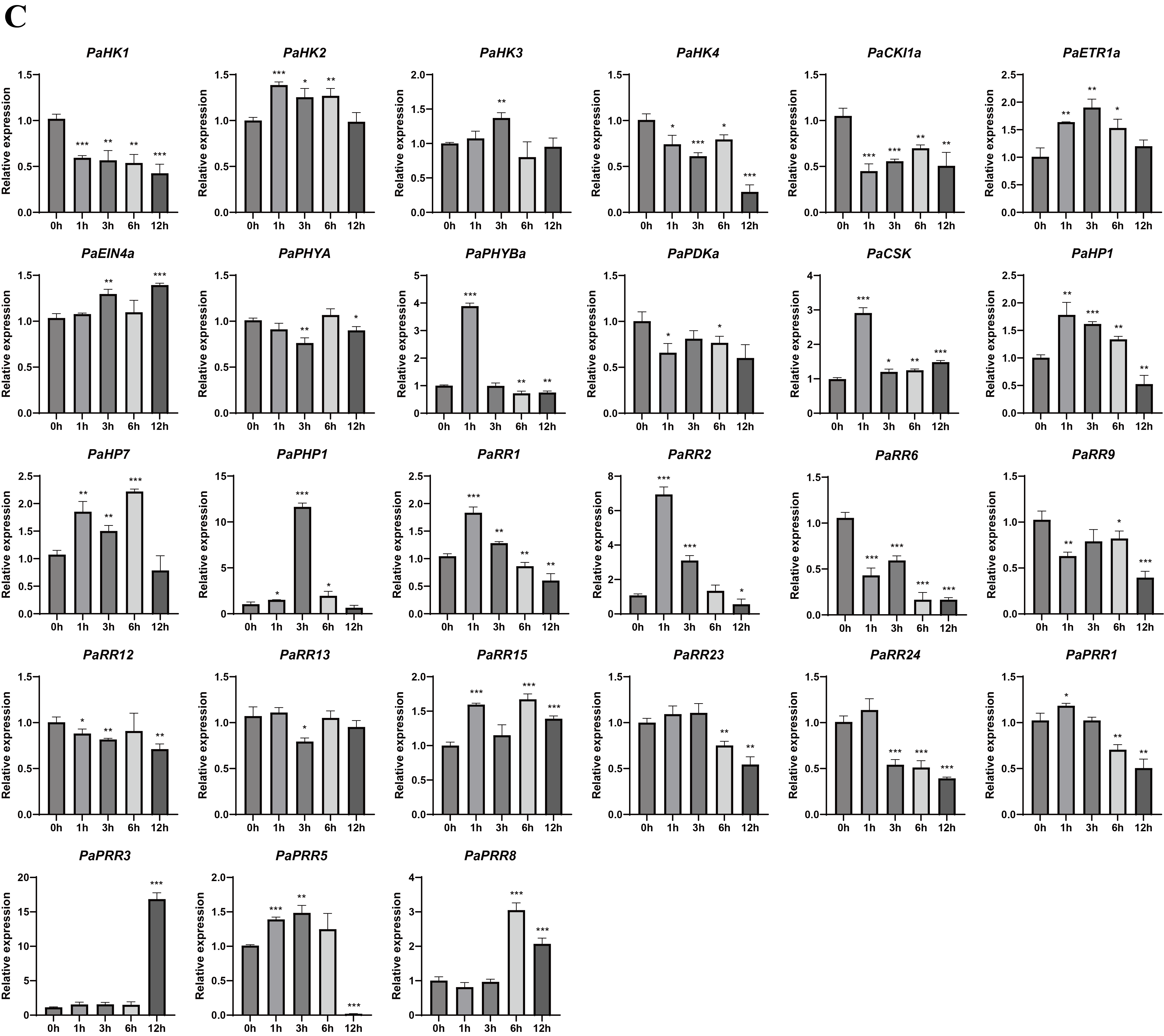


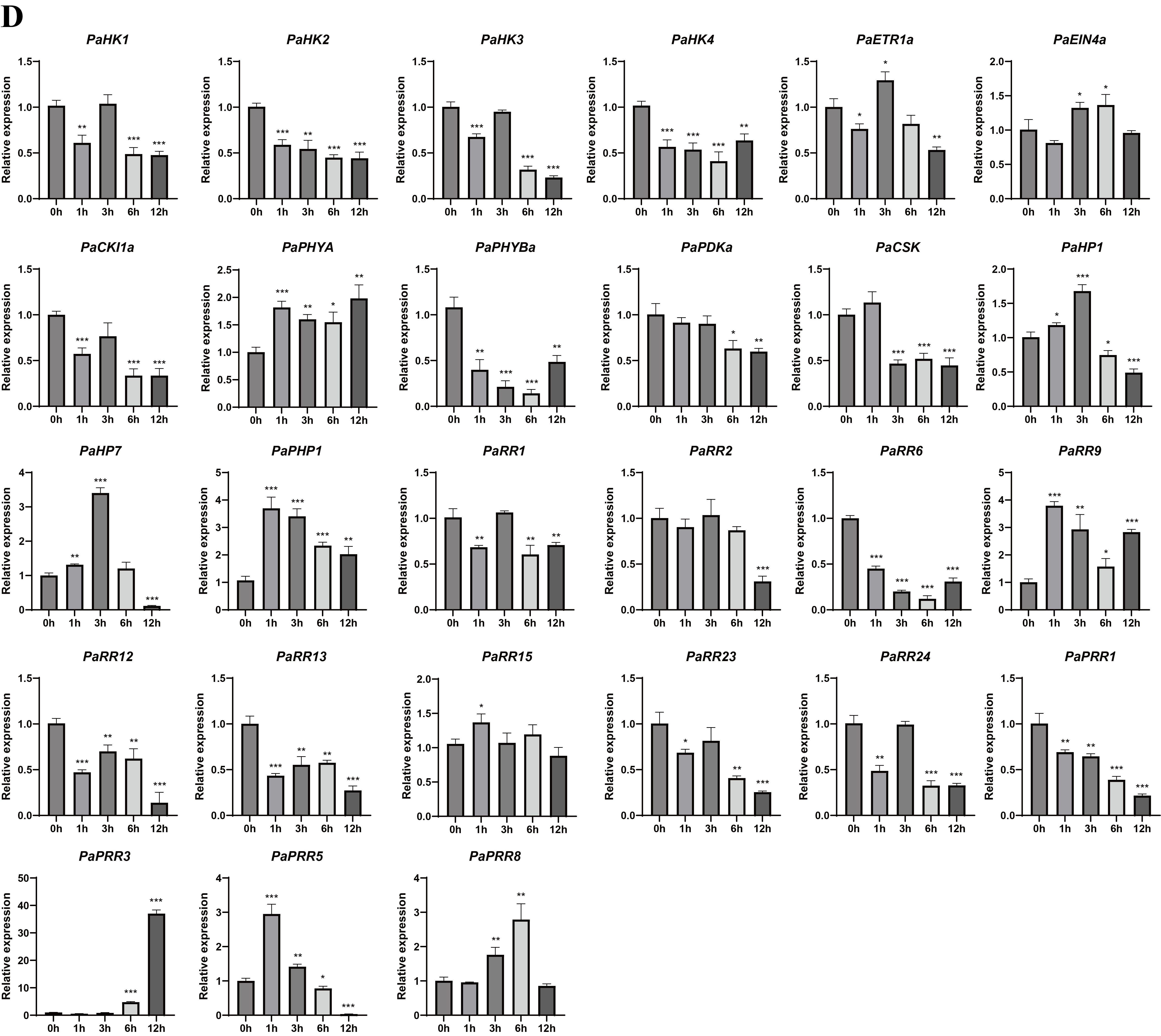


**Figure S6.** The expression patterns of petunia TCS genes in response to hormone (tZ (A) and ABA (B)) and stresses (drought (C) and salt (D)). The second true leaves were collected at 0, 1, 3, 6, and 12 h after the treatments. Asterisks on the top of bars (SD values) indicate statistically significant difference between the compared pairs (**p* < 0.05; ***p* < 0.01; ****p* < 0.001).
